# Learning causal networks with latent variables from multivariate information in genomic data

**DOI:** 10.1371/journal.pcbi.1005662

**Published:** 2017-10-02

**Authors:** Louis Verny, Nadir Sella, Séverine Affeldt, Param Priya Singh, Hervé Isambert

**Affiliations:** 1 Institut Curie, PSL Research University, CNRS, UMR168, Paris, France; 2 Sorbonne Universités, UPMC Univ Paris 06, Paris, France; Microsoft Research, UNITED STATES

## Abstract

Learning causal networks from large-scale genomic data remains challenging in absence of time series or controlled perturbation experiments. We report an information- theoretic method which learns a large class of causal or non-causal graphical models from purely observational data, while including the effects of unobserved latent variables, commonly found in many genomic datasets. Starting from a complete graph, the method iteratively removes dispensable edges, by uncovering significant information contributions from indirect paths, and assesses edge-specific confidences from randomization of available data. The remaining edges are then oriented based on the signature of causality in observational data. The approach and associated algorithm, miic, outperform earlier methods on a broad range of benchmark networks. Causal network reconstructions are presented at different biological size and time scales, from gene regulation in single cells to whole genome duplication in tumor development as well as long term evolution of vertebrates. Miic is publicly available at https://github.com/miicTeam/MIIC.

## Author summary

The reconstruction of causal networks from genomic data is an important but challenging problem. Predicting key regulatory interactions or genomic alterations at the origin of human diseases can guide experimental investigation and ultimately inspire innovative therapy. However, causal relationships are difficult to establish without the possibility to directly perturb the organisms’ genome for ethical or practical reasons. Besides, unmeasured (latent) variables may be hidden in many genomic datasets and lead to spurious causal relationships between observed variables. We propose in this paper an efficient computational approach, miic, that overcomes these limitations and learns causal networks from non-perturbative (observational) data in the presence of latent variables. In addition, we assess the confidence of each predicted interaction and demonstrate the enhanced robustness and accuracy of miic compared to alternative existing methods. This approach can be applied on a wide range of datasets and provide new biological insights on regulatory networks from single cell expression data or genomic alterations during tumor development. Miic is implemented in an R package freely available to the scientific community under a General Public License.

## Introduction

Network reconstruction methods have become ubiquitous to analyze large-scale information-rich data from the latest genomic technologies. Recently, methodological advances in the field have been seeking to learn causal relationships using time series or controlled perturbation experiments [1, 2]. However, such strategies can be technically impracticable or costly, if not unethical, in many biological contexts.

Alternatively, graphical models can be learned by simply observing enough random variations in unperturbed data, as for the reconstruction of gene regulatory networks from single-cell gene expression data. However, most methods based on this principle, such as Bayesian search-and-score [3], sparse inverse covariance estimation [4], maximum entropy [5] or diffusion map [6] methods, assume as underlying models either causal networks with only directed edges or non-causal networks with only undirected edges. Thus, they cannot uncover nor rule out causality in observational data. By contrast, constraint-based methods [7–10], which identify structural constraints corresponding to all dispensable edges in a graph, can in principle uncover causality from purely observational data. Advanced constraint-based methods [9, 10] reconstruct Markov equivalent models of a broad class of “ancestral graphs” [11], that include undirected (−), directed (→) and possibly bidirected (↔) edges originating from latent common causes, *L*, unobserved in the available data (*i.e.* ⇠ *L* ⇢). However, constraint-based methods are often not robust on small datasets and have algorithmic complexity issues when including unobserved latent variables [9–12]. Yet, latent variables are commonly found in many real applications, as in the case of an unobserved transcription factor *TF* co-regulating two co-expressed genes, *i.e.* *G*_1_ ⇠ *TF* ⇢ *G*_2_ (see example of single cell transcriptomics in the Results section). These unobserved variables should not be ignored in practice, as they actually impact the causal relationships between observed variables, leading to spurious causal association between co-regulated genes *G*_1_ and *G*_2_ in the previous example. While the algorithmic difficulties of constraint-based methods have so far limited their applicability in practice, understanding cause-effect relationships [13] remains of primary interest to model complex biological systems and anticipate their response to environmental changes or genetic alterations.

We report here an information-theoretic method, that simultaneously circumvents the complexity and robustness issues of constraint-based approaches, and demonstrate its applicability to real biological data. The method builds on an earlier information- theoretic approach [14], in order to *i)* include latent variables, a notorious conceptual and algorithmic difficulty in causal network reconstruction [9–13], and *ii)* provide an edge specific confidence assessment of retained edges, which lacks in traditional constraint-based methods. Both aspects are important in practice to reconstruct robust networks from actual biological data. The approach is applied to reconstruct causal networks from a variety of genomic datasets at different biological size and time scales, from single cells to organisms and entire phyla.

## Results

### Background: Signature of causality and unobserved latent variables in observational data

Our information-theoretic method for network reconstruction is based on the analysis of multivariate information [14–19], *I*(*X*; *Y*; *Z*; ⋯), which extends the concept of mutual information [20] beyond two variables, *I*(*X*; *Y*) = ∑_*x*,*y*_ *p*(*x*,*y*)log(*p*(*x*,*y*)/*p*(*x*)*p*(*y*)), where *p*(*x*), *p*(*y*) and *p*(*x*,*y*) are the measured probability distributions of single or joint variables *X* and *Y* from the available data D (see Materials and methods). Most importantly, unlike two-point mutual information, *I*(*X*; *Y*), which cannot distinguish causal from non-causal relations between variables *X* and *Y*, multivariate information involving more than two points, *I*(*X*; *Y*; *Z*; ⋯), may imply cause-effect relationships between the underlying variables, S1 File.

**Fig 1:**
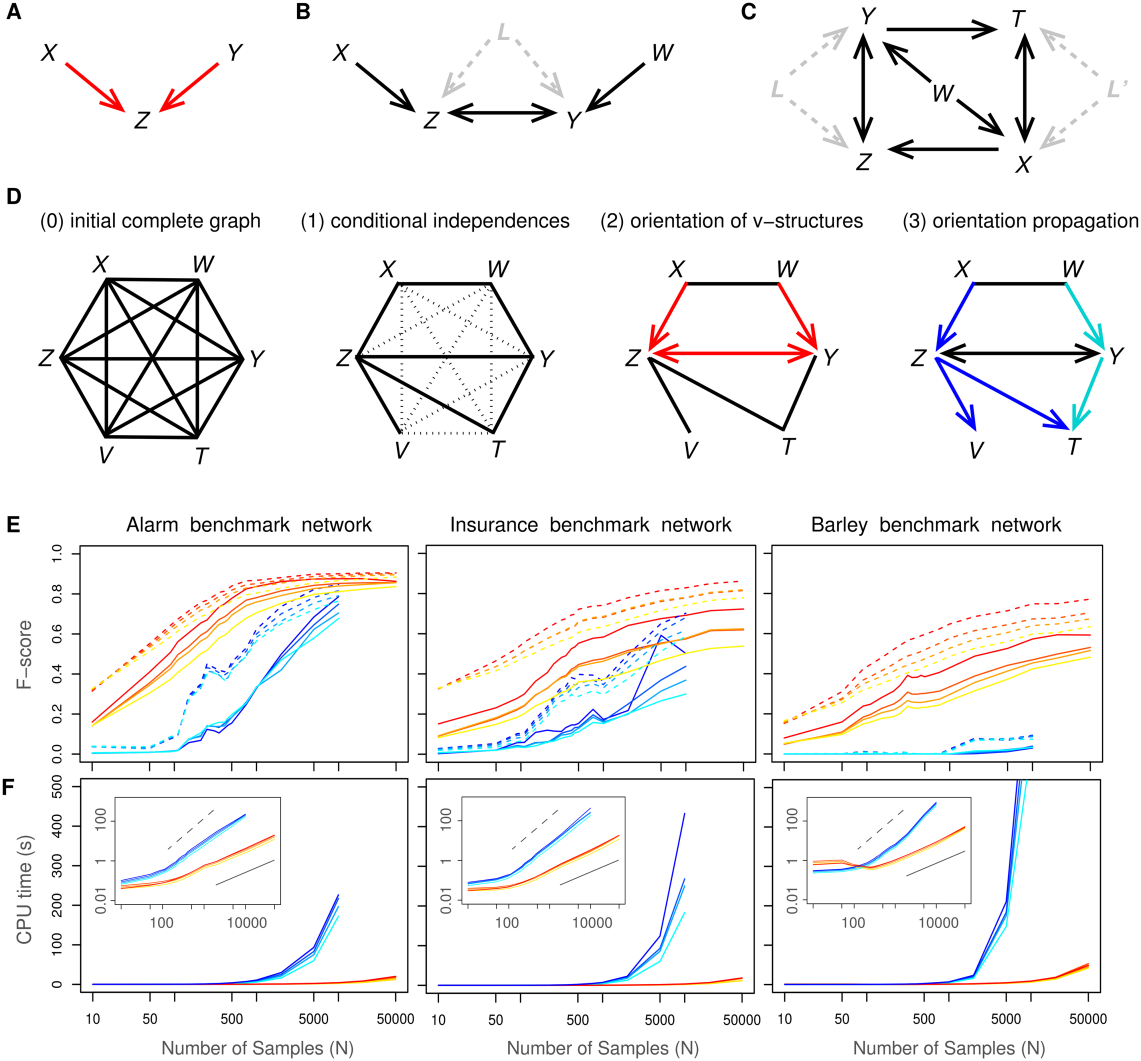
Learning causal networks with latent variables. (**A**) A v-structure. (**B**) Bidirected edges in collider paths indicate the presence of latent common cause(s), *L*, unobserved in the dataset. (**C**) Conditional independence in the presence of latent variables requires to be conditioned on non-adjacent variables, in general [9, 10], such as for the pair {*Z*,*T*} which needs to be conditioned on *X*, *Y* and non-adjacent *W*, *I*(*Z*; *T*|*X*,*Y*,*W*) = 0, as one cannot condition on the unobserved latent variables, *L* or *L*′, *e.g.* *I*(*Z*; *T*|*X*,*L*) = 0 or *I*(*Z*; *T*|*Y*,*L*′) = 0. (**D**) Outline of the successive steps of constraint-based approaches (see also Algorithm steps in Materials and methods). (**E**) F-score (harmonic mean of Precision and Recall, S1, S2 and S3 Figs) of miic algorithm (warm colors) for 0%, 5%, 10% and 20% of latent variables (top to bottom curves), compared to the RFCI algorithm [10] (cold colors) on benchmark networks of increasing complexity disregarding (dashed lines) or including (solid lines) edge orientations: Alarm [37 nodes, avg. deg. 2.5, 509 parameters], Insurance [27 nodes, avg. deg. 3.9, 984 parameters] and Barley [48 nodes, avg. deg. 3.5, 114,005 parameters]. (**F**) Computation times of miic (warm colors) compared to RFCI (cold colors). Inserts: computation times in log scale showing a linear scaling (solid bar) in the limit of large datasets, *τ_cpu_* ∼ *N*^1±0.1^, with miic, and a close to quadratic scaling (dashed bar), *τ_cpu_* ∼ *N*^1.8±0.3^, with RFCI.

In fact, the signature of causality in purely observational data is associated to a unique correlation pattern involving at least three variables [13, 21]: it concerns two mutually (or conditionally) independent variables, *I*(*X*; *Y*) = 0, which are therefore not connected to each other, yet both connected to a third variable *Z*, Fig 1A. This situation entails the orientations of a ‘v-structure’ or ‘unshielded’ collider, *X* → *Z* ← *Y*, because the edges *XZ* and *YZ* cannot be undirected, nor *Z* be a cause of *X* or *Y*, as these alternative graphical models imply correlations that would contradict independence between *X* and *Y*. V-structures are the hallmark of causality in observational data: networks with v-structures are necessary causal, while causal models without v-structures can be shown to be equivalent to their undirected counterparts from the viewpoint of observational data.

Beyond v-structures, colliders may also be found in series along a collider path, as in *X* → *Z* ↔ *Y* ← *W*, Fig 1B & 1C, where the bidirected edge, *Z* ↔ *Y*, indicates that *Z* is not a cause of *Y* nor *Y* a cause of *Z*. It implies that the correlation between *Z* and *Y* is due to at least one latent common cause, *L*, unobserved in the available dataset, *Z* ⇠ *L* ⇢ *Y*, as outlined above. Hence, statistical dependencies and independencies in purely observational data can, in principle, provide structural constraints for network reconstruction as well as information on causal relationships across observed and possibly unobserved latent variables. These results underline the wealth of information which cannot be captured from two-point correlations only.

### An information-theoretic method to learn causal networks with latent variables

The signature of causality and unobserved latent variables in multi-point correlation statistics enables to rephrase constraint-based methods [7–10] within an information-theoretic framework. Constraint-based approaches, sketched in Fig 1D, start from a fully connected network and proceed by iteratively removing dispensable edges between variables *X* and *Y* for which a conditional independence can be found, *i.e.* *I*(*X*; *Y*|{*A*_*i*_}) = 0 (Fig 1D, step 1). This rationale of constraint-based methods can be interpreted from an information perspective [22], using the generic decomposition of mutual information, *I*(*X*; *Y*), relative to the set of variables {*A*_*i*_},

$$\begin{array}{c} I\left(X;Y\right)=I\left(X;Y;\left\{A_{i}\right\}\right)+I\left(X;Y|\left\{A_{i}\right\}\right), \end{array}$$ (1)

where *I*(*X*; *Y*;{*A*_*i*_}) can be seen as the global indirect contribution of {*A*_*i*_} to *I*(*X*; *Y*) and *I*(*X*; *Y*|{*A*_*i*_}) as the remaining (direct) contribution (see Eq 8 in Materials and methods). Conditional independence, *I*(*X*; *Y*|{*A*_*i*_}) = 0, then implies that {*A*_*i*_} is a ‘separation set’ which intercepts all indirect paths contributing to the total mutual information, *i.e.* *I*(*X*; *Y*) = *I*(*X*; *Y*; {*A*_*i*_}). In practice, however, conditional mutual information cannot be exactly zero for finite datasets but the probability that the *XY* edge should be removed can be estimated from the available data as, *P*_*XY*_ ∼ exp(−*NI*(*X*; *Y*|{*A*_*i*_})), up to some normalization constant, where *N* is the number of independent samples (S1 File). The undirected network ‘skeleton’, resulting from the removal of all dispensable edges, is then partially directed by orienting all v-structures (Fig 1D, step 2), based on the signature of causality, outlined above, and propagating these orientations on downstream edges (Fig 1D, step 3), based on specific propagation rules consistent with ancestral graphs [23].

**Fig 2:**
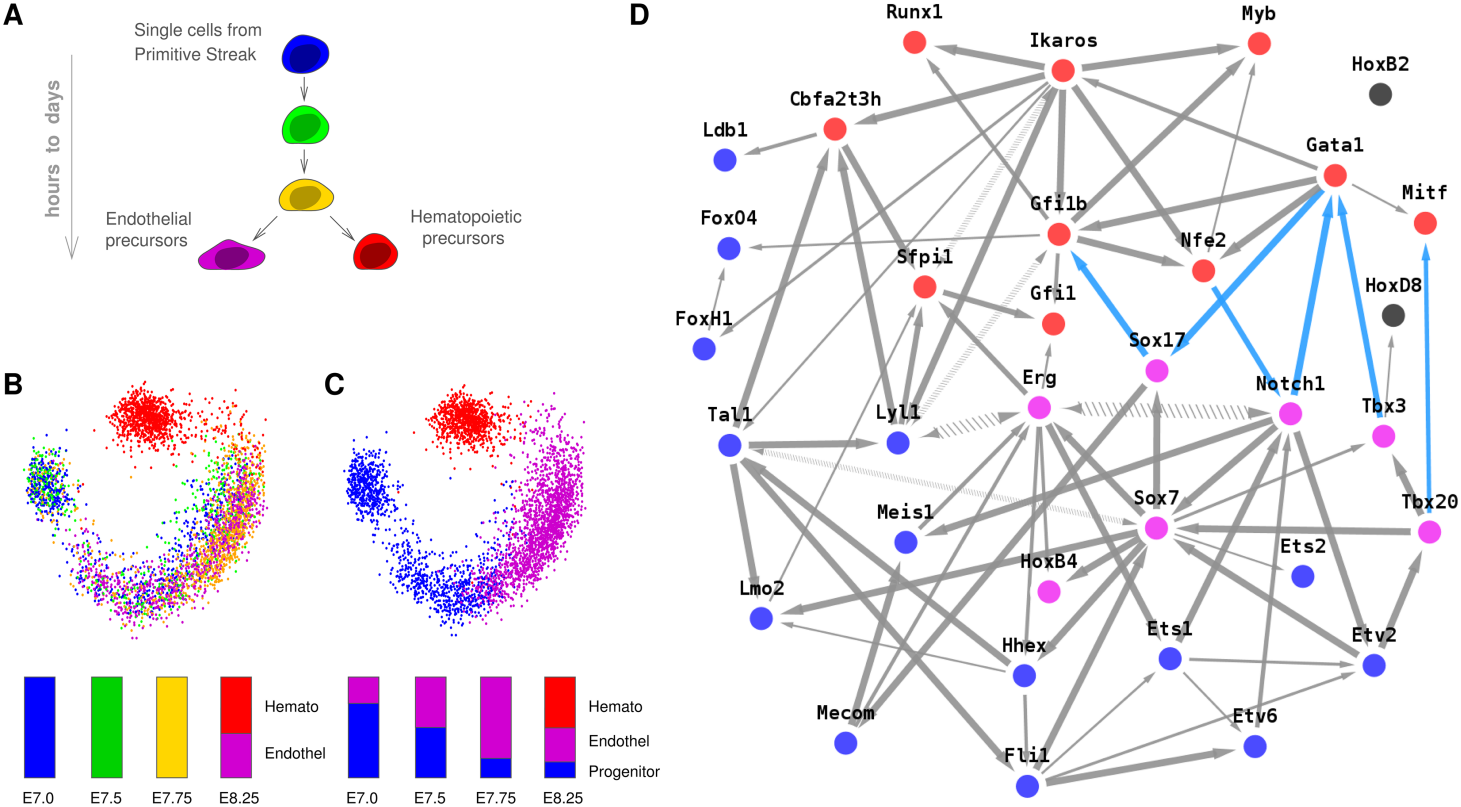
Network reconstruction at cellular level. (**A**) Hematopoietic / endothelial differentiation in single cells from mouse embryos [24]. (**B**) Principal component analysis and (**C**) K-means clustering of gene expression data [24] with histograms showing the relative proportions of cell populations at each data point (E7.0 to E8.25). (**D**) Hematopoietic / endothelial differentiation regulatory network between hematopoietic specific (red), endothelial (violet), common (blue) and unclassified (gray) TFs. Graph predicted with miic R-package and visualized using cytoscape (blue edges correspond to repressions).

The main computational complexity of constraint-based methods is to uncover a valid combination of contributing nodes {*A*_*i*_} for each dispensable edge *XY*. In absence of latent variables, the combinatorial search can be restricted to the sole neighbors of *X* or *Y*, which are sufficient to intercept all information contributions from indirect paths [7, 8]. However, this efficient algorithm cannot be used in the presence of latent variables, as collider paths may require to extend the combinatorial search for conditioning set {*A*_*i*_} to non-adjacent variables of *X* and *Y* [9], as illustrated in Fig 1C. In practice, this intrinsic difficulty stemming from latent variables has been addressed through more complex algorithmic approaches, such as the FCI algorithm [9] and its more recent approximate variant, RFCI [10]. Beyond algorithmic complexity issues, traditional constraint-based methods are also known to be highly sensitive to sampling noise inherent to finite datasets and are not robust on typical datasets of interest (*e.g.* expression data of 30 to 40 genes measured in a few hundreds to thousands of single cells [24], see application and Fig 2 below).

The present algorithmic approach, miic (multivariate information-based inductive causation), circumvents the complexity and robustness issues of standard constraint-based methods by avoiding to directly tackle the combinatorial search of complete separation sets. Instead, it progressively collects, one-by-one, their most likely contributors, {*A*_*i*_}_*n*_ = {*A*_1_, *A*_2_, ⋯, *A*_*n*_}, based on a quantitative score for each pair of variables *XY* (S1 File). The global indirect contribution is then obtained iteratively as,

$$\begin{array}{c} I\left(X;Y;{\left\{A_{i}\right\}}_{n}\right)=I\left(X;Y;{\left\{A_{i}\right\}}_{n-1}\right)+I\left(X;Y;A_{n}|{\left\{A_{i}\right\}}_{n-1}\right), \end{array}$$ (2)

where *I*(*X*; *Y*; *A*_*n*_|{*A*_*i*_}_*n*−1_) > 0, corresponds to the contribution of the most likely *n*th variable *A*_*n*_ after collecting the first *n*−1 most likely contributors, {*A*_*i*_}_*n*−1_ (see Eq 10 in Materials and methods). We demonstrate in the current study that this iterative framework, which proved to be robust to sampling noise in absence of latent variables [19], can in fact be extended to include latent variables by collecting the contributors {*A*_*i*_} within the whole set of observed variables, instead of amongst the sole neighbors of *X* and *Y* in absence of latent variables [14]. This simple approach to include latent variables circumvents the algorithmic complexity of standard constraint-based methods [9, 10], while improving ten to hundred folds their performance in both prediction accuracy and running time, as discussed in the next section.

### Algorithmic performance on causal and non-causal benchmark datasets

We have assessed the performance of miic on a broad range of causal and non-causal benchmark networks from real-life as well as simulated datasets from *P* ≃ 30 up to 500 variables and *N* = 10 up to 50,000 independent samples (Materials and methods). The causal benchmark networks, which include an increasing fraction (0% to 20%) of hidden latent variables, are derived using partially observed Bayesian networks, that is, considering some variables as hidden. These unobserved variables are usually present in many real applications and cannot be ignored in practice, as they actually impact the causal relationships between observed variables, as illustrated in Fig 1B–1D. The non-causal benchmark datasets have been obtained from Monte Carlo sampling of Ising-like interacting networks sharing approximately the same two-point direct correlations with real-life benchmark causal networks but lacking causality. Monte Carlo sampling leads, however, to significant correlations between successive samples, which needs to be taken into account through an effective number of independent samples (Materials and methods).

Reconstructed causal networks have been compared to *partial ancestral graphs* (PAGs) [23], which are the representatives of the Markov equivalent class of all ancestral graphs consistent with the conditional independences in the available data. In practice, benchmark PAGs have been derived by hiding some variables in benchmark directed acyclic graphs (DAG) using the dag2pag function of the pcalg package with slight modifications [25, 26]. The alternative inference methods used for comparison with miic are the FCI algorithm [9] and its recent approximate variant RFCI [10] implemented in the pcalg package [25, 26]. The results obtained with FCI and RFCI are in fact very similar and we only present here comparisons with the more recent RFCI algorithm [10]. RFCI’s results are shown for an adjustable significance level *α* = 0.01 and using the *stable* implementation of the skeleton learning algorithm, as well as the *majority rule* for the orientation and propagation steps [27], which give overall the best results. The results have been evaluated in terms of running time, as well as, Precision (or positive predictive value), Recall or Sensitivity (true positive rate), and F-score, which is the harmonic mean of Precision and Recall (Materials and methods). Precision, Recall and F-score have been derived for the undirected skeleton of the networks (dashed lines in Fig 1E) or taking into account edge orientations (solid lines in Fig 1E).

The results on benchmark networks are presented in Fig 1E and 1F, as well as S1, S2, S3, S4, S5, S6 and S7 Figs. Miic outperforms classical constraint-based approaches, including its advanced approximate variant RFCI, Fig 1E, especially on networks with many underlying parameters. It achieves significantly better or comparable results with much fewer samples (Fig 1E, S1, S2 and S3 Figs), and is typically ten to hundred times faster (Fig 1F). In addition, miic’s ability to learn complex ancestral networks, which require conditioning on non-adjacent variables, can be directly demonstrated on the example of Fig 1C network, S4 Fig. The complexity of miic algorithm, while difficult to evaluate exactly, proves to be linear in terms of sample size (Fig 1F) and quadratic in terms of network size for sparse graphs irrespective of the inclusion of latent variables (S5 Fig). By contrast, traditional constraint-based methods exhibit roughly quadratic complexity in terms of sample size (Fig 1F) and much steeper complexity scaling in terms of network size, especially when latent variables are included [12]. Furthermore, no causality is predicted by miic for non causal datasets, even from small effective numbers of independent samples (Materials and methods and S6 and S7 Figs). This underlines miic accuracy to uncover true causality.

### Edge confidence assessments

This information-theoretic method and its algorithmic implementation (S1 Software) are very general and can be applied to a wide range of datasets, provided a sufficient number of independent samples is available. We report here the results obtained with genomic datasets spanning a broad range of biological size and time scales from single cells and tissues to organisms and entire phyla. In addition to including latent causal variables, we have also assessed the confidence of predicted edges with an edge specific confidence ratio $C_{XY}=P_{XY}/\langle P_{XY}^{\;\text{rand}}\rangle$, where *P*_*XY*_ is the probability to remove the *XY* edge, introduced above, and $\langle P_{XY}^{\;\text{rand}}\rangle$ the average of the same probability after randomizing the datasets for each variable (see Materials and methods, and S1 File section 2.2 for details). Hence, the lower *C*_*XY*_, the higher the confidence on the *XY* edge, which can be used to retain only high confidence edges in the predicted networks.

**Fig 3:**
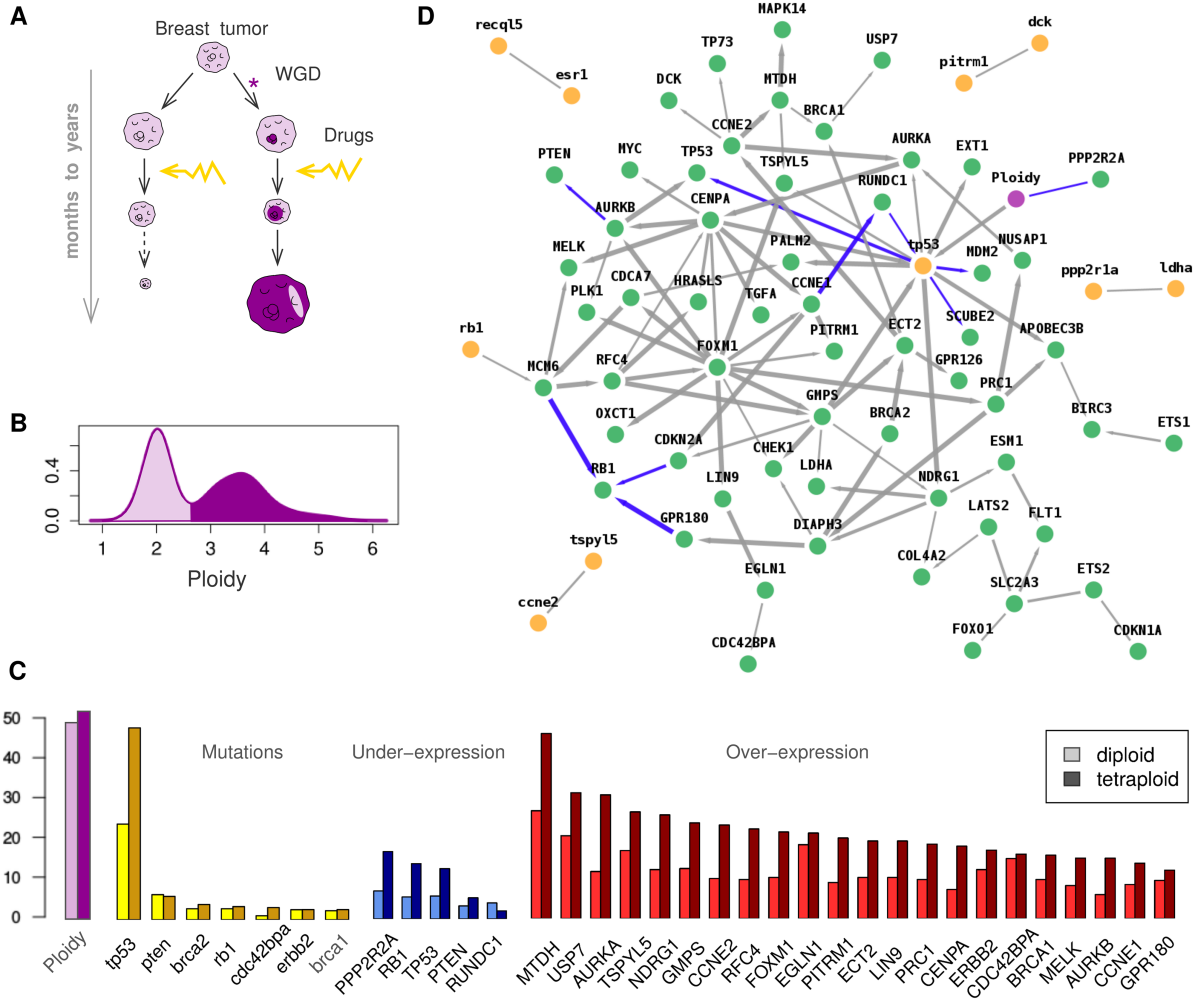
Network reconstruction at tissue level. (**A**) Tumor development and drug resistance in the presence of tetraploid tumor cells following whole genome duplication (WGD). (**B**) Ploidy distribution in the 807 tumor samples and (**C**) genomic alterations: ploidy, mutations, normalized under-expression and over-expression changes from COSMIC database [34]. (**D**) Genomic alteration network obtained between average ploidy (violet), gene mutations (yellow, lower case) and under- or over-expressions (green, upper case). Graph predicted with miic R-package and visualized using cytoscape (blue edges correspond to repressions).

**Fig 4:**
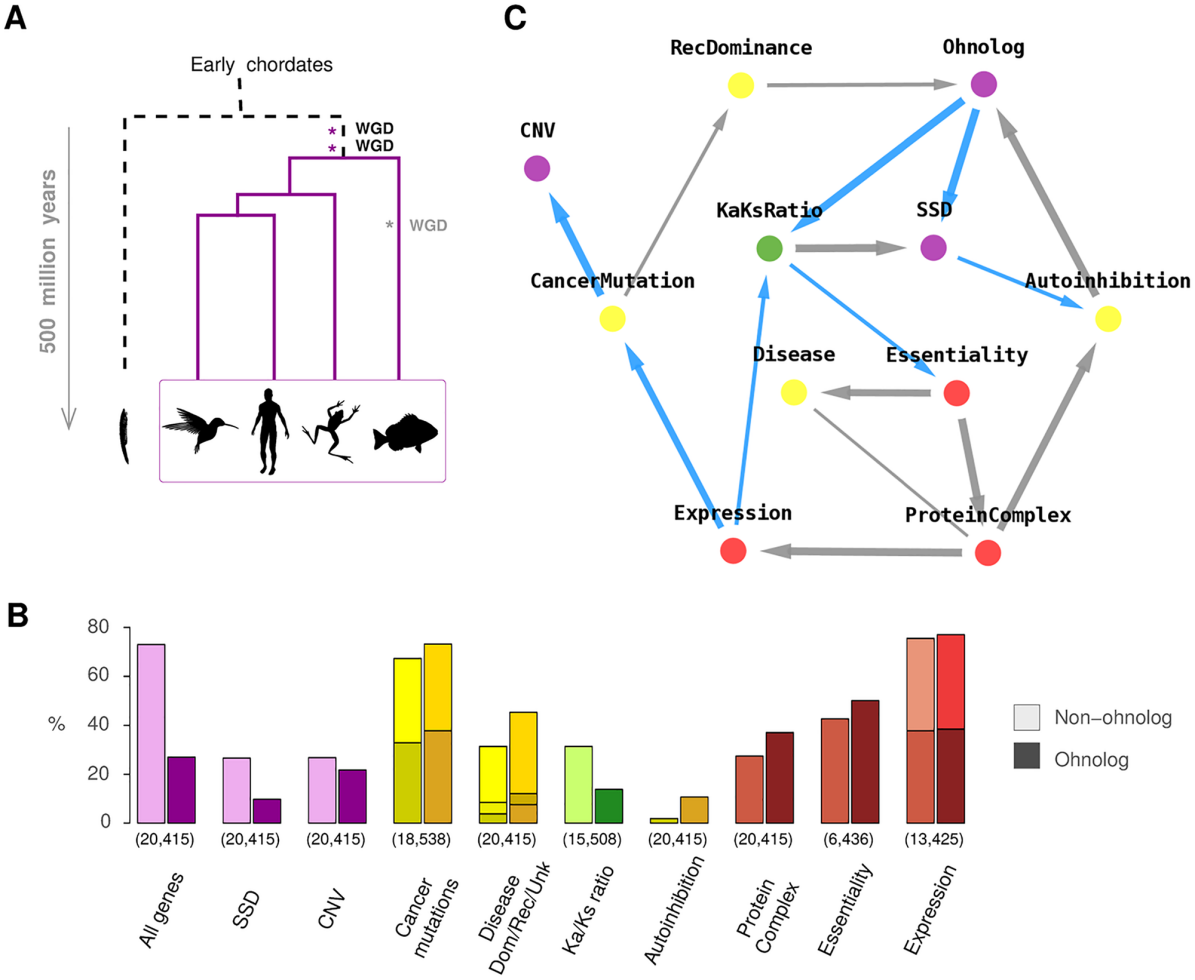
Network reconstruction at organismal and phylogenetic levels. (**A**) Two rounds of whole genome duplication (WGD) have led to the evolutionary radiation of vertebrates (and similarly with a third 300-MY-old WGD in teleost fish). (**B**) Biased distributions of genomic properties within ‘non-ohnolog’ and ‘ohnolog’ genes retained from WGDs in early vertebrates [45]. Numbers in brackets indicate the numbers of genes for which each property is identified, Materials and Methods and S1 Data. (**C**) Genomic property network of human genes, see main text. Graph predicted with miic R-package and visualized using cytoscape (blue edges correspond to repressions).

Interestingly, the effect of confidence filtering on the reconstruction of benchmark networks (S8 & S9 Figs) demonstrates that the filtering of individual edges improves the Precision of the reconstruction (at the expense of its Sensitivity or Recall) not only for the network skeleton, as expected, but also for the network orientations, while retaining overall similar F-scores. This demonstrates the interest and consistency of using such confidence filtering to obtain an enhanced and tunable precision of the reconstructed networks for real biological applications. Indeed, an enhanced precision might be desirable in many practical applications for which the correctness of predicted edges is more important than the occasional dismissal of less certain edges. All network reconstructions presented in Figs 2, 3 & 4 have been obtained with an edge specific confidence *C*_*XY*_ < 10^−3^, while network skeletons obtained before edge filtering are displayed in S11, S14 and S15 Figs.

The general three-step reconstruction scheme of miic (*i.e.* Step 1- graph skeleton, Step 2- edge filtering, Step 3- edge orientation) is also sensitive to the fine tuning of other algorithmic parameters such as the complexity criterion introduced to estimate finite size effects. All results presented in this paper have been obtained with the decomposable Normalized Maximum Likelihood (NML) criterion introduced in [28, 29], which was shown to yield significantly better results than more traditional BIC/MDL criterion on benchmark networks, especially on small datasets, leading to simultaneous improvements in both recall and precision [19]. Choosing the BIC/MDL instead of NML criterion in the three genetic network applications, Figs 2, 3 & 4, leads to somewhat sparser reconstituted networks including 82% to 100% of initial edges, yet no additional edges (*i.e.* consistent with a lower recall), and 66% to 75% conserved edge orientations (*i.e.* identical ‒, →, ← and ↔ edges), see S1 Table.

### Analysis of expression data in single cells

At cellular level, we reconstructed regulatory networks from single cell expression data at the time of endothelial and hematopoietic differentiations from the primitive streak cells of the mouse early embryo, Fig 2A. This concerns the formation of primitive erythroid cells, a distinct and transient red blood cell lineage arising directly from mesodermal progenitors with restricted hematopoietic potential [32], by contrast to the highly studied definitive erythroid cells which arise from multipotent hematopoietic stem cells.

The dataset for this application is from Moignard *et al* [24] and includes the expression of 33 transcription factors (TFs) along with 13 non-TF genes (markers) in 3,934 single cells extracted at 4 different times of the mouse embryo development (days E7.0, E7.5, E7.75 and E8.25), Fig 2A–2C and S10 Fig. The cells extracted from E8.25 were also divided by the authors in two different pools: potential endothelial precursors and potential hematopoietic precursors based on the expression of the *Runx1* hematopoietic marker. Gene expression was collected using single cell qRT-PCR and binarized by the authors, leading to two-state (on / off) expression levels in the available dataset. Pooling all cells together regardless of their developmental timing (from day E7.0 to E8.25), we first analyzed their population heterogeneity using principal component analysis (PCA), Fig 2B, and K-means clustering, Fig 2C. Three main cell populations are identified and can be interpreted, based on gene functional classification (Materials and methods), as progenitor, endothelial precursor and hematopoietic precursor populations, whose relative proportions vary from E7.0 to E8.25, Fig 2C.

The network predicted by miic, Fig 2D, includes 75 edges with *C*_*XY*_ < 10^−3^ out of 82 edges in the unfiltered skeleton, S11 Fig. The differentiation bifurcation between endothelial and hematopoietic precursors, seen through principal component (Fig 2B) and clustering (Fig 2C) analyses, also clearly appears in the reconstructed regulatory network, Fig 2D, after labelling hematopoietic specific TFs (in red), endothelial TFs (in purple) and common TFs expressed in both precursor lineages (in blue), Materials and Methods. In fact, most predicted regulatory interactions across lineage specific TFs correspond to regulatory inhibitions (in blue), which might originate either from direct regulatory repressions or possibly through indirect ‘ancestor’ regulations involving unobserved intermediary TFs. In addition, a number of known regulatory interactions are correctly predicted in the inferred network, Fig 2D, such as *Ikaros → Gfi1b* and *Ikaros → Lyl1* [31], *Tal1 → Fli1* and *Tal1 → Lmo2* [32] as well as *HoxB4 → Erg* (with opposite orientation) and *Sox7 → Erg* [24]. Yet, there are also many predicted regulations in miic network that have not been reported so far as well as a number of regulations documented in definitive erythroid cells [32] that appear to be missing in primitive erythroid cells (*e.g.* *Est1 → Tal1*, *Sfpi1 → Tal1* and *Sfpi1 → Myb*). These results suggest a number of testable predictions, including five bidirected edges consistent with the absence of direct regulations reported between these genes. Indeed, bidirected edges imply the necessity to invoke unobserved latent co-regulators between such genes. In particular, the unmeasured *Gata2* expression is possibly implicated in the co-regulation of *Erg ↔ Lyl1*, based on an earlier study [33]. Hence, beyond the consistency with earlier reports as well as testable predictions, miic results may also help pinpoint possible latent regulators unobserved in Moignard *et al*’s study [24], such as regulators specific to the initial progenitor cells, not yet committed to either hematopoietic or endothelial lineages and accounting for about 70% of analyzed cells at day E7.0, Fig 2C.

### Analysis of genomic and ploidy alterations in breast tumors

At tissue and organismal levels, we analyzed genomic alterations on breast tumors from the online Catalog of Somatic Mutations in Cancer (COSMIC) datase [34], Fig 3A–3C.

The dataset, which contains 807 samples without predisposing *BRCA1/2* germline mutations, includes somatic mutations (from whole exome sequencing) and expression level information for 91 genes. These 91 genes have been selected based on earlier studies on mutation and/or expression alterations in breast cancer, Materials and Methods. Gene non-synonymous mutation status is binarized (yes / no) and gene expression status is categorized as under-, normal- or over-expressed by the COSMIC database. S12 Fig provides the distribution of altered expressions and S13 Fig the distribution of mutations for the 91 genes of interest. In addition to gene mutations and altered expression levels, we also integrated information on sample average ploidy, provided by the COSMIC database (release v76) and discretized the clearly bimodal ploidy distribution (Fig 3B) with ploidy < 2.7 considered as diploid cells and ≥ 2.7 taken as tetraploid cells, in agreement with COSMIC convention [34]. Among the 807 samples, 401 correspond to diploid tumoral cells and 398 to tetraploid tumoral cells (8 samples have no ploidy information). As expected, *TP53, RB1* and *PTEN* tumor suppressors tend to be mutated, downregulated or lost, especially in tetraploid tumors, Fig 3B & 3C, which also exhibit significant normalized expression alterations, Fig 3C.

The network predicted by miic is shown Fig 3D. We first note that, due to the limited numbers of samples (N = 807) and recurrent gene mutants (Fig 3C and S13 Fig), most gene mutations are not confidently linked to any altered expression levels (compare Fig 3D with edge confidence *C*_*XY*_ < 10^−3^ to the unfiltered skeleton, S14 Fig), with the notable exceptions of *TP53* and *RB1* mutations, which have a significant impact on gene expressions, Fig 3D. Interestingly, the overall effect of tetraploidization on normalized gene expression, Fig 3C, is predicted to be largely indirect and mediated by *TP53* mutations which lead to dysregulation of mitosis controling genes, such as the under-expression of *PPP2R2A* [35] and over-expression of *AURKA* and *CENPA* genes. In addition, tetraploidy and *TP53* mutations tend also to be concomitant with over-expression of metabolic (*GMPS*) and cell-growth modulating genes (*TSPYL5*, *NDRG1* and *FOXM1*) [36], favoring tumor progression and metastasis, as well as higher expression of *APOBEC3B*, which promotes mutational heterogeneity within tumors and, thereby, their drug resistance through subclonal selection [37]. Hence, miic results provide a direct link between the long-known incidence of *TP53* mutations in (breast) cancer and the tetraploidization of tumor cells. These results, supported by a number of recent reports [35, 37–40], shed light on the poor prognosis associated with tetraploid tumors and their resistance to chemotherapy [40]. This presumably occurs as tetraploid cells can exploit their genome redundancy and heterogeneity to evolve resistance strategies under drug treatments, Fig 3A.

Interestingly, this dynamics of tetraploid tumors in the course of cancer progression and treatment echoes the success of tetraploid species in the course of eukaryote evolution. Indeed, genome doubling events, possibly associated to environmental changes, have repeatedly led to successful evolutionary radiations of biodiverse subphyla, such as the vertebrates and the flowering plants [41], although the underlying selection mechanism has remained a matter of debate [41–44].

### Analysis of two rounds of tetraploidization in vertebrate evolution

We have investigated with miic this long term evolution following the two rounds of tetraploidization that occurred in early vertebrates some 500 million years ago, Fig 4A. While long lost species and subphyla cannot be directly studied, the genetic make up of extant vertebrates provides an information-rich data on the selection processes at work since these ancient genome duplications. In particular, we aimed at identifying the genomic properties potentially responsible for the biaised retention of ‘ohnolog’ gene duplicates [45] retained from these genome duplications in early vertebrates.

We obtained 20,415 protein-coding genes in the human genome from Ensembl (v70) and collected information on the retention of duplicates originating either from the two whole genome duplications at the onset of vertebrates (‘ohnolog’) or from subsequent small scale duplications (‘SSD’) as well as copy number variants (‘CNV’), Fig 4B and S1 Data [45]. 5,504 ohnolog genes retained from the two rounds of whole genome duplications (WGDs) in the common vertebrate ancestor were obtained from the ‘Ohnologs’ server based on multi-species comparison of synteny [45]. All the small scale duplicates (SSDs) in the human genome were obtained from Ensembl Compara using BioMart [46], and were restricted to the 4,506 genes duplicated after the WGDs. Genes with copy number variants (CNVs) were obtained from the Database of Genomic Variants [47]. A total of 5,185 genes were identified to be CNV genes as their entire coding sequence fell within one of the CNV regions in this database.

We then collected information on the genomic properties of these 20,415 human genes, including their sequence conservation (‘Ka/Ks ratio’), protein autoinhibitory folds and participation to protein complexes, their expression levels across tissues, association with dominant or recessive diseases and susceptibility to cancer mutations as well as their essentiality for development and reproduction, see Materials and methods.

The resulting causal network, predicted by miic, relates the origin of duplicated genes in the human genome (*i.e.* ‘ohnolog’, SSD or CNV gene duplicates) to their genomic properties and association to diseases, Fig 4C. The reconstructed network implies that the retention of ohnolog duplicates is more directly linked to their susceptibility to dominant mutations and protein autoinhibitory folds than other genomic properties such as dosage balance constraints in protein complexes [42], gene essentiality or expression levels, which do not exhibit direct links to ohnolog retention, Fig 4C, even on the network skeleton obtained before edge confidence filtering, S15 Fig. Hence, miic analysis based on observational data provides an independent confirmation as well as significant extension of earlier reports based on correlations between two or three genomic properties [43] and on simple population genetic models [48]. All together, these results support an evolutionary retention of ohnologs by purifying selection through dominant diseases in tetraploid species (consistent with the retention of ohnologs with low Ka/Ks ratio, Fig 4C, indicating sequence conservation) while small scale duplicated genes have been retained through positive selection (consistent with their higher Ka/Ks ratio, Fig 4C, indicative of underlying adaptation).

## Discussion

We report in this paper a novel information-theoretic method that learns a broad class of network models including latent causal effects from purely observational data, that is, in absence of time series or controlled intervention experiments, which can be technically impractical, costly or unethical to obtain in many biological contexts.

The methodology of our approach is quite general and follows a three-step scheme:

- Step 1- Find a graph skeleton taking into account latent variables.
- Step 2- Remove weakly supported edges based on a confidence criterion.
- Step 3- Determine edge orientations based on the signature of causality.

While resembling traditional constraint-based methods such as FCI, miic is in fact designed to be much faster and more robust to finite sample size through greedy algorithmic strategies based on quantitative information-theoretic scores at each algorithmic step, *i.e.* Step 1: iterative collection of most likely contributors based on an contributor ranking scheme, Step 2: filtering of weakly supported edges through an edge-specific confidence assessment, and Step 3: successive orientation of the remaining edges based on decreasing orientation probabilities.

Unlike earlier robust methods for network reconstruction [3–6], this general scheme circumvents the need to choose between causal and non-causal graphical models *a priori*, as the most appropriate class of models is directly learned from the available data. In addition, the approach can uncover the effect of unobserved latent variables, a notorious conceptual and algorithmic difficulty in causal network reconstruction [13]. Yet, latent variables are usually present in many real applications and cannot be ignored in practice, as they actually impact the causal relationships between observed variables.

More specifically, miic relies on the analysis of multivariate information [14–19], which extends the concept of mutual information to more than two variables. In practice, miic integration of constraint-based methods within an information-theoretic framework leads to greatly improved performances in both prediction accuracy (Fig 1E) and running time (Fig 1F) as well as favorable scalings in terms of sample size (Fig 1F) and network size (S5 Fig). The likelihood ratio formalism also enables to derive an edge specific confidence index, *C*_*XY*_, which allows to filter predicted edges to obtain an enhanced and tunable precision of the reconstructed networks. This might be desirable in many applications for which the correctness of predicted edges is more important than the occasional dismissal of less certain edges.

We have used miic to reconstruct causal networks from a variety of genomic datasets at different biological size and time scales, from gene regulation in single cells (Fig 2) to whole genome duplication in tumor development (Fig 3) as well as long term evolution of vertebrates (Fig 4). In all these applications, miic provides testable predictions and new biological insights summarized below:

1. on the hematopoietic / endothelial differentiation network (Fig 2), miic results shed lights on the regulatory interactions in primitive erythropoietic differentiation for which much less is known compared with definitive erythropoiesis [30]. We predict, in particular, the central role of regulators such as *Ikaros* in the hematopoietic precursor population, and *Sox7* and *Erg* in the endothelial precursor population, as well as the causal effects of unobserved latent variables such as the transcription factor *Gata2*;
2. on the development of breast cancer, miic network reconstruction (Fig 3) highlights the direct association between tetraploidization and *TP53* mutations, by contrast with earlier studies on non-cancerous cell lines [40, 49] but in agreement with findings on actual tumors and their resistance to treatments [38, 40]. These results are also consistent with the high incidence of tetraploid tumors in patients with *BRCA1/2* germline mutations [50];
3. finally, concerning the impact of whole genome duplications in vertebrate evolution, miic results (Fig 4) refute the general view in the field on the retention of ohnologs through dosage balance constraints [42]. Instead, miic multivariate analysis demonstrates the role of dominant deleterious effects on the retention of ohnologs, which significantly extends and confirms earlier reports based on correlations between two or three genomic properties [43, 44] and independent population genetic results based on first-principles evolutionary models [48].

Beyond the three genomic network reconstructions presented in this paper (Figs 2, 3 and 4), we anticipate that this information-theoretic approach may help uncover cause-effect relationships in other information-rich datasets from different fields of biological interest, such as developmental biology, neuroscience, clinical data analysis and epidemiology. The causal network learning tool, miic, is implemented in an R-package software with open source code and freely available under a General Public License (S1 Software).

## Materials and methods

### Application

#### Gene functional classification in hematopoiesis/epithelial differentiation

The early hematopoiesis single cell transcription data come from Moignard *et al.*, 2015 [24]. The expression of 33 TFs and 13 non-TF genes (markers) have been obtained by single cell qRT-PCR and binarized (on/off) by the authors. The 33 TFs can be classified into 3 categories related to their function, using the Mouse Genome Database [34] as well as the TF expressions at the different time points in the original experiment [24]:

- “Hematopoietic”: This group gathers the TFs for which we found a function in hematopoietic differentiation, without finding any evidence of a role in endothelium formation in the litterature. The corresponding genes linked to hematopoietic function are: *Eto2, Sfpi1/PU.1, Runx1, Nfe2, Myb, Mitf, Ikaros, Gfi1b, Gfi1, Gata1*.
- “Endothelial”: For these genes, the main function found in the litterature is in endothelial development. The corresponding genes linked to endothelial function are: *Ets2, Erg, Tbx3, Tbx20, Sox7, Sox17, Notch1, HoxB4*.
- “Common”: These TFs have been shown to be involved in both hematopoietic and endothelial differentiation. The corresponding genes linked to both hematopoietic and endothelial functions are: *Fli1, Etv6, Etv2, Ets1, Tal1, Meis1, Mecom, Lyl1, Lmo2, Ldb1, Hhex*.

#### Signature gene set in breast cancer progression

The choice of specific genes for monitoring genomic alterations has been guided by earlier studies and breast cancer-specific molecular tests [51], which demonstrate that altered expression profiles can reveal patient overall outcome [52]. In particular, the MammaPrint genomic assay relies on a 70-gene expression profile to assess patient breast cancer recurrence risk [52]. This signature classifies patient either as high-risk or low-risk for long-term development of distant metastasis. The relevance of the MammaPrint 70-gene profile has already been assessed by multiple studies, *e.g.* [52, 53]. Interestingly, although the MammaPrint biomarker genes were selected from a completely data-driven approach, they are enriched with specific cancer hallmarks [54] acquired in the course of tumorigenesis and metastasis progression [55].

In this study, we investigated the interrelations between ploidy, mutation and expression level alterations for 91 genes in breast tumors. Specifically, we first considered the mutation status and expression levels of 50 genes out of the 70 Mammaprint biomarkers for which a hallmark of cancer has been identified [55]. We also considered 18 commonly altered genes in breast cancer (*ERBB2, ESR1, TP53, RB1, MYC, JUN, CDKN2A, BCL2, APOBEC3B, PTEN, MDM2, USP7, UBE3A, SPDYE7P, PLK1, BAX, MET, FOXM1*) [56]. In addition, 23 genes related to ploidy alteration were also included (*TP73, LATS2, MAPK14, CDKN1A, CHEK1, AURKB, AURKA, BRCA1, BRCA2, DUSP5, MST1, PPP1R13L, BIRC3, TGFA, ETS1, ETS2, HIF1A, LDHA, FOXO1, NDRG1, PPP2R1A, PPP2R2A, CCNE1*) [38, 40].

#### Genomic properties of ohnolog genes in vertebrates

The genomic properties susceptible to be associated with the retention of ‘ohnolog’ gene duplicates (as well as SSD and CNV duplicates) in the human genome have been obtained from various resources:

- **Cancer mutations**. Cancer mutation profiles for all the protein coding genes were obtained from the COSMIC database [34]. We counted all the non-synonymous mutations per unit length in all the available samples, and partitioned the 18,538 genes with available mutation information into three equal frequency bins (S1 Data).
- **Disease genes**. Human disease genes were collected from OMIM, GeneCards [57], and from published curated lists [44, 58] and combined to give a total of 7,171 disease genes.
- **Recessive** ***vs*** **dominant genes**. Based on the inheritance information from Online Mendelian Inheritance in Man (OMIM) database, we could obtain 981 and 952 genes that were described as autosomal dominant and autosomal recessive genes respectively.
- **Autoinhibition**. Genes with autoinhibitory protein folds were obtained from search and manual curation in PubMed and in various databases (OMIM, SwissProt, NCBI Gene and GeneCards). Additional autoinhibitory candidates with the domains known to be frequently implicated in autoinhibition (*e.g.* SH3, DH, PH, CH, Drf and Eth domains) were obtained based on the domains identified using HMMER search [59] against Pfam database [60]. This led to a total of 881 genes with autoinhibitory protein folds (S1 Data).
- **Essentiality**. A total of 6,436 1-to-1 mouse orthologs obtained using BioMart and tested for lethality or infertility phenotypes on loss-of-function or knockout mutations in mouse were obtained from the Mouse Genome Informatics database [32]. 2,729 [resp. 3,227] of these 6,436 genes were found to be essential [resp. non-essential] genes in mouse.
- **Protein complex**. A total of 6,119 genes involved in protein complex formation were obtained by combining the protein complexes from Human Protein Reference Database [61], CORUM database [62], the human soluble protein complex census [63], and the human genes belonging to the Gene Ontology term “protein complex” under Cellular Component.
- **Ka/Ks ratio**. We obtained Ka/Ks (or dN/dS) ratios between human and amphioxus (*Branchiostoma floridae*) orthologs using the KaKs_Calculator 2.0 [64]. Ka/Ks ratios were retrieved for a total of 15,508 genes and partitioned into 75% lower ratio < 0.2 (*i.e.* more conserved sequences) and 25% higher ratio ≥ 0.2 (*i.e.* rapidly evolving sequences)
- **Expression levels**. Gene expression levels for 78 healthy human tissues and cell types [65] were downloaded from BioGPS [66]. Affimetrix tags were mapped to Ensembl gene IDs using BioMart and annotation provided by BioGPS. Expression levels from different tags for the same gene were averaged after removing the tags that bind to multiple genes. A total of 13,425 genes with an expression level were partitioned into three equal frequency bins based on the their median expression across 78 tissues/cell types.

These genomic properties susceptible to be associated with the retention of ‘ohnolog’, SSD and CNV gene duplicates are provided as S1 Data.

For each genomic property or combination of properties for which a number of samples presents missing data, multivariate information, such as *I*(*X*; *Y*|{*A*_*i*_}), are computed on the number of available samples *N*_*a*_ without missing data for *X*, *Y* and {*A*_*i*_} variables (*N*_*a*_ < *N*). Finite size corrections are then estimated based on *N*_*a*_ instead of *N* samples (S1 File). This assumes that the missing data is missing completely at random.

### Methodology

#### Ancestral graphs

The miic software reconstructs Markov equivalent models of the broad class of ‘***ancestral graphs***’ [11] which can contain three types of edges, undirected (−), directed (→) and bidirected (↔) edges, but:

1. no directed cycles (*i.e.* *X* → → ⋯ → → *Y* with *X* ← *Y*)
2. no almost directed cycles (*i.e.* *X* → → ⋯ → → *Y* with *X* ↔ *Y*)
3. no arrowheads pointing to an undirected edge (*i.e.* → − or ↔ −)

#### Multivariate information and most likely information contributors

The miic algorithm is an information-theoretic method that learns graphical models by progressively uncovering the information contributions of indirect paths in terms of ***multivariate information***.

The ***multivariate information*** between *p* variables, *I*(*X*_1_; ⋯; *X*_*p*_), is defined through alternating (inclusion-exclusion) sums of multivariate entropies *H*({*X*_*i*_}) = −∑_{*x*_*i*_}_ *p*({*x*_*i*_})log *p*({*x*_*i*_}) over all subsets of variables {*X*_*i*_} ⊆ {*X*_1_,⋯,*X*_*p*_} as [15–17],

$$\begin{array}{ccc} I(X_{1};\cdots ;X_{p})\;\;\; & = & \;\;\;\sum_{i}H\left(X_{i}\right)-\sum_{i<j}H\left(X_{i},X_{j}\right)+\sum_{i<j<k}H\left(X_{i},X_{j},X_{k}\right)-\cdots \\ & & \;\;\;{\left(-1\right)}^{k-1}\;\;\;\;\sum_{i_{1}<\cdots <i_{k}}\;\;\;\;H\left(X_{i_{1}},\cdots ,X_{i_{k}}\right)+\cdots {\left(-1\right)}^{p-1}H\left(X_{1},\cdots ,X_{p}\right)\;\;\; \end{array}$$ (3)

In particular, for *p* = 2 and 3 variables, it yields,

$$\begin{array}{ccc} I(X;Y) & \;\;\;\;=\;\;\;\; & H(X)+H(Y)-H(X,Y) \end{array}$$ (4)

$$\begin{array}{ccc} I(X;Y;A) & \;\;\;\;=\;\;\;\; & H(X)+H(Y)+H(A)-H(X,Y)-H(X,A)-H(Y,A)+H(X,Y,A)\;\;\;\; \end{array}$$ (5)

where the 3-point information, *I*(*X*; *Y*; *A*), can be positive or negative unlike the 2-point (mutual) information, *I*(*X*; *Y*), which is always positive [20]. Conditional multivariate information, *I*(*X*_1_; ⋯; *X*_*p*_|*A*), are defined similarly as multivariate information, *I*(*X*_1_; ⋯; *X*_*p*_), but in terms of conditional multivariate entropies [18], *H*({*X*_*i*_}|*A*). In particular, conditional mutual information is defined as,

$$\begin{array}{ccc} I(X;Y|A) & = & H(X|A)+H(Y|A)-H(X,Y|A)\\ & = & -H(A)+H(X,A)+H(Y,A)-H(X,Y,A) \end{array}$$ (6)

using the definition of conditional entropy [20], *H*(*X*|*A*) = *H*(*X*,*A*) − *H*(*A*). Then combining the expressions of *I*(*X*; *Y*|*A*) and *I*(*X*; *Y*; *A*) yields a generic decomposition rule relative to a variable *A* or a set of variables {*A*_*i*_}_*m*_ = {*A*_1_,*A*_2_,⋯,*A*_*m*_} as,

$$\begin{array}{ccc} I(X;Y) & = & I(X;Y|A)+I(X;Y;A) \end{array}$$ (7)

$$\begin{array}{ccc} I(X;Y) & = & I\left(X;Y|{\left\{A_{i}\right\}}_{m}\right)+I\left(X;Y;{\left\{A_{i}\right\}}_{m}\right) \end{array}$$ (8)

and conditioning Eq 7 on {*A*_*i*_}_*n*−1_ and setting *A* ≡ *A*_*n*_ yields,

$$\begin{array}{ccc} I(X;Y|{\left\{A_{i}\right\}}_{n-1}) & = & I\left(X;Y|{\left\{A_{i}\right\}}_{n}\right)+I\left(X;Y;A_{n}|{\left\{A_{i}\right\}}_{n-1}\right) \end{array}$$ (9)

which can be combined with Eq 8, setting {*A*_*i*_}_*m*_ = {*A*_*i*_}_*n*−1_ or {*A*_*i*_}_*n*_, to yield the following iterative scheme on the contribution increment of the collected set {*A*_*i*_}_*n*_ (see Results),

$$\begin{array}{ccc} I(X;Y;{\left\{A_{i}\right\}}_{n}) & = & I\left(X;Y;{\left\{A_{i}\right\}}_{n-1}\right)+I\left(X;Y;A_{n}|{\left\{A_{i}\right\}}_{n-1}\right) \end{array}$$ (10)

As explained in S1 File, only positive information terms, *I*(*X*; *Y*; *A*_*n*_|{*A*_*i*_}_*n*−1_)>0, contribute to the global mutual information between *X* and *Y* through the iterative decomposition of Eq 9,

$$\begin{array}{c} I\left(\;X;Y\;\right)=I\left(\;X;Y;A_{1}\;\right)+I\left(\;X;Y;A_{2}|A_{1}\;\right)+\cdots +I\left(\;X;Y;A_{n}|{\left\{A_{i}\right\}}_{n-1}\;\right)+I\left(\;X;Y|{\left\{A_{i}\right\}}_{n}\;\right) \end{array}$$ (11)

where the most likely contributors *A*_*n*_ after collecting the first *n*−1 contributors {*A*_*i*_}_*n*−1_ is chosen by maximizing *I*(*X*; *Y*; *A*_*n*_|{*A*_*i*_}_*n*−1_) > 0, while taking into account the finite size *N* of the dataset (S1 File). The approach provides also a natural ranking of the edges *XY* of the graph, *R*(*XY*; *A*_*n*_|{*A*_*i*_}_*n*−1_), based on the likelihood of their best next contributor *A*_*n*_ (Eq. S20 in S1 File).

By contrast, negative information, *I*(*X*; *Y*; *A*_*n*_|{*A*_*i*_}_*n*−1_) < 0, do not contribute to *I*(*X*; *Y*) but are the signature of causality in observational data and are used to orient v-structures, such as *X* → *A*_*n*_ ← *Y* (S1 File).

#### Description of miic algorithmic pipeline

The implementation of the information-theoretical approach miic proceeds in three steps corresponding to the following algorithmic pipeline, Fig 1D (S1 File):

- Step 1: *Learning skeleton taking into account latent variables*
  Starting from a fully connected undirected graph, miic iteratively removes all dispensable edges after collecting one-by-one their most likely contributors {*A*_*i*_} based on the edge ranking order, *R*(*XY*; *A*_*n*_|{*A*_*i*_}_*n*−1_) (Eq. S20 in S1 File), and using the following pseudocode,
  **Repeat**: take the top edge *XY* with highest rank *R*(*XY*; *A*_*n*_|{*A*_*i*_}_*n*−1_):
  – Update its contributor list: {*A*_*i*_}_*n*_ ← {*A*_*i*_}_*n*−1_ + *A*_*n*_
  – If *I*(*X*; *Y*|{*A*_*i*_}_*n*_) is not significant (given the finite number *N* of samples): remove edge *XY*
  – Else: Search for the next best contributor *A*_*n*+1_ of edge *XY* (if one exists with *I*(*X*; *Y*; *A*_*n*+1_|{*A*_*i*_}_*n*_) > 0) and update the ranking order *R*(*XY*; *A*_*n*+1_|{*A*_*i*_}_*n*_)
  **Until**: no more edges can be removed
- Step 2: *Confidence estimate and sign of retained edges*
  Once a first skeleton has been obtained using Step 1, the confidence on each retained edge can be estimated through an edge specific confidence ratio *C*_*XY*_ based on the probability *P*_*XY*_ ∼ exp(−*NI*(*X*; *Y*|{*A*_*i*_})) to remove a directed edge *X* → *Y* from the graph G (S1 File),
  $$\begin{array}{ccc} C_{XY} & = & \frac{P_{XY}}{\left\langle P_{XY}^{\;\text{rand}}\right\rangle } \end{array}$$ (12)
  where $\langle P_{XY}^{\;\text{rand}}\rangle$ is the average of the probability to remove the *XY* edge after randomly permutating the dataset for each variable. Hence, the lower *C*_*XY*_, the higher the confidence on the *XY* edge. We favor the confidence estimate *C*_*XY*_ based on likelihood ratios (Eq. S21 in S1 File) to the alternative confidence estimate based on p-value, which corresponds to the probability that $P_{XY}^{\;\text{rand}}\leq P_{XY}$ over random permutations. Indeed, p-value estimates require much more random permutations than *C*_*XY*_ estimates for strong edges with *NI*(*X*; *Y*|{*A*_*i*_}) ≫ 1, as virtually all random permutations correspond to $P_{XY}^{\;\text{rand}}>P_{XY}$ in that case, leading to under-estimated p-values ≃ 0.
  In addition, the sign of each retained edge, *X* − *Y*, is defined by the sign of the partial correlation coefficient, *ρ*_*XY*⋅*A*_, between *X* and *Y* conditioned on its derived contributors *A* = {*A*_*i*_} in Step 1, with positive edges corresponding to positive partial correlations and negative edges corresponding to negative partial correlations, *i.e.* partial anti-correlations (S1 File).
- Step 3: *Probabilistic orientation and propagation of remaining edges*
  Given the skeleton obtained from Step 1, possibly filtered through Step 2, initially unspecified endpoint marks (∘) can be established, as arrow tail (−) or head (>), following probabilistic orientation and propagation rules of unshielded triples 〈*X*, *Y*, *Z* 〉_*X*⌿*Y*_, S1 File (where * below stands for any endpoint mark),
  **Repeat**: take the top 〈*X*, *Y*, *Z* 〉_*X*⌿*Y*_ with highest endmark orientation / propagation probability
  – If *I*(*X*; *Y*; *Z*|{*A*_*i*_}_*n*_) < 0 and X* − ∘*Z*∘ −*Y or X* → *Z*∘ − *Y, orient edge(s) to form a v-structure X* → *Z* ← *Y
  – Else If *I*(*X*; *Y*; *Z*|{*A*_*i*_}_*n*_) > 0 and X* → *Z*∘ − ∘*Y* or X* → *Z*∘ →*Y*, Propagate second edge direction to form a non-v-structure X* → *Z* → *Y*
  **Until**: no additional endmark orientation / propagation probability >1/2

#### Algorithmic performance on benchmark networks with latent variables

The performance of the information-theoretic method miic was tested on benchmark ancestral graphs with latent variables using partially observed real-life networks (*i.e.* considering some variables as hidden) as well as random networks generated with the causal modeling tool Tetrad V (http://www.phil.cmu.edu/tetrad). Reconstructed networks are compared to *partial ancestral graphs* (PAGs) [23], which are the representatives of the Markov equivalent class of all ancestral graphs consistent with the conditional independences in the available data. In practice, benchmark PAGs have been derived by hiding some variables in benchmark directed acyclic graphs (DAG) using the dag2pag function of the pcalg package with slight modifications [25, 26]. PAGs have been generated for an increasing fraction (0% to 20%) of randomly picked latent variables having a significant topological effect on the underlying network (*i.e.* excluding parentless vertices with a single child or vertices without child).

The results are evaluated in terms of skeleton Precision (or positive predictive value), *Prec* = *TP*/(*TP* + *FP*), Recall or Sensitivity (true positive rate), *Rec* = *TP*/(*TP* + *FN*), as well as F-score = 2 × *Prec* × *Rec*/(*Prec* + *Rec*) for increasing sample size from *N* = 10 to 50,000 data points. We also define additional Precision, Recall and F-scores taking into account the edge endpoint marks of the predicted networks against the corresponding benchmark PAGs. This amounts to label as false positives, all true positive edges of the skeleton with different arrowhead endpoint marks (*i.e.* arrowhead (>) *versus* tail or undefined (−/∘) endpoint marks) as the PAG reference, *TP*_misorient_, leading to the orientation-dependent definitions *TP*′ = *TP* − *TP*_misorient_ and *FP*′ = *FP* + *TP*_misorient_ with the corresponding PAG Precision, Recall and F-scores taking into account arrowhead endpoint marks.

The alternative inference methods used for comparison with miic are the FCI algorithm [9] and its recent approximate variant RFCI [10] implemented in the pcalg package [25, 26]. The results obtained with FCI and RFCI are in fact very similar and we only present here comparisons with the more recent RFCI algorithm [10]. RFCI’s results are shown for an adjustable significance level *α* = 0.01 and using the *stable* implementation of the skeleton learning algorithm, as well as the *majority rule* for the orientation and propagation steps [27], which give overall the best results.

For each sample size (*N* = 10 to 50,000) and fraction of hidden variables (0% to 20%), miic and RFCI inference methods have been tested on 20 combinations of hidden variables and 50 dataset replicates each. S1, S2 and S3 Figs give the average results over these multiple combinations of latent variables and dataset replicates and compare the reconstructed networks including orientations (solid lines) or without orientation (*i.e.* skeleton, dashed lines) to the theoretical PAG (or its skeleton) of the benchmark network.

#### Algorithmic performance on undirected benchmark networks

The performance of miic was also tested on non-causal benchmark networks reconstructed from Monte Carlo sampling of Ising-like interacting systems.

To this end, real-life causal networks, such as Alarm and Insurance, have been transformed into non-causal Ising-like networks (with binary spin variables *x*_*i*_ = ±1) by setting pairwise interacting parameters *k*_*ij*_ between connected variables *X*_*i*_ and *X*_*j*_, so as to approximately reproduce the pairwise conditional mutual information *I*(*X*_*i*_; *X*_*j*_|***A***_*X*_*i*_*X*_*j*__) of the original real-life causal network. This yields benchmark networks sharing approximately the same two-point direct correlations with the original causal networks but lacking causality, as the couplings *k*_*ij*_ between spins are all symmetric by construction.

One million configurations of these Ising-like interacting systems have been generated using Monte Carlo sampling approach. It consists in flipping a fraction of the spins randomly and accepting each newly generated configuration with probability, min (1, exp(−Δ*E*_*k*_)), where Δ*E*_*k*_ = *E*_*k*+1_ − *E*_*k*_, is the interacting energy difference between successive configurations, $E_{k}=-\sum_{i<j}^{\text{edges}}k_{ij}x_{i}x_{j}$. The fraction of spins randomly flipped (∼10%) has been ajusted to ensure that about half of the newly generated configurations are accepted at each Monte Carlo iteration, in order to efficiently sample configuration space. This leads, however, to significant correlations between successive accepted configurations with a roughly exponential decay between *n* distant samples, *C*(*n*) ≃ *C*(0)exp(−*n*/*R*) = *C*(0)*α*^*n*^, where $C\left(n\right)=C\left(k-\ell \right)=\langle \sum_{i}\delta x_{i}^{\left(\ell \right)}\delta x_{i}^{\left(k\right)}\rangle$ is the average autocorrelation with lag between the *k*th and ℓth samples (with *n* = *k*−ℓ), where $\delta x_{i}^{\left(k\right)}=x_{i}^{\left(k\right)}-\bar{x_{i}}$.

The effective number of independent samples $N_{\text{eff}}^{*}$ can then be estimated through the apparent increase of variance between the *N* partially correlated samples as [67],

$$\begin{array}{ccc} V_{N} & = & \frac{1}{N^{2}}\sum_{k}\sum_{\ell }\left\langle \sum_{i}\delta x_{i}^{\left(k\right)}\delta x_{i}^{\left(\ell \right)}\right\rangle \\ & = & \frac{1}{N^{2}}\sum_{k}\sum_{\ell }C\left(k-\ell \right)\\ & = & \frac{1}{N}[C\left(0\right)+2(1-\frac{1}{N})C\left(1\right)+2(1-\frac{2}{N})C\left(2\right)+\cdots +\frac{2}{N}C\left(N-1\right)] \end{array}$$ (13)

which leads for a first order Markov process with *C*(*n*) = *C*(0)*α*^*n*^ to,

$$\begin{array}{ccc} V_{N} & = & \frac{C(0)}{N}[1+2(1-\frac{1}{N})\alpha +2(1-\frac{2}{N})\alpha^{2}+\cdots +\frac{2}{N}\alpha^{N-1}]\\ & ≃ & \frac{C(0)}{N}\frac{1+\alpha }{1-\alpha }=\frac{C(0)}{N_{\text{eff}}^{*}} \end{array}$$ (14)

yielding a smaller effective number of samples $N_{\text{eff}}^{*}<N$ for correlated datasets (*α* > 0) as,

$$\begin{array}{ccc} N_{\text{eff}}^{*} & = & N\frac{1-\alpha }{1+\alpha } \end{array}$$ (15)

This estimate suggests to use $N_{\text{eff}}^{*}$, instead of *N*, to compute the finite size corrections of the miic approach, in order to correct for the correlations between successive samples generated through Monte Carlo sampling. Yet, as the presence of correlations between successive samples is *a priori* incompatible with the requirement of independent samples in the maximum likelihood framework, we have first assessed miic performance over the full range of possible effective sample size, *i.e.* 0 < *N*_eff_/*N* ≤ 1, for *N* = 1,000 to 300,000 successive samples from the one-million-long sample series.

The results are shown in S6 Fig and S6 Fig in terms of Precision, Recall, F-score and Fraction of (wrongly) directed edges for the Alarm-like and Insurance-like undirected networks.

The nearly exponential decay of the autocorrelation function for Alarm-like (S6 Fig, *R* = 7.758, *α* = 0.872) and Insurance-like (S6 Fig, *R* = 7.676, *α* = 0.87) undirected networks leads to very close values for the predicted effective number of samples for these graphs according to Eq 15, $N_{\text{eff}}^{*}/N≃0.068-0.069$.

Interestingly, we found that the F-score, which is a trade-off between optimizing Precision and Recall, reaches a maximum for all sample sizes (*N* = 1,000 to 300,000) around the predicted effective number of samples, that is when $N_{\text{eff}}/N=N_{\text{eff}}^{*}/N≃0.069$, see vertical dashed lines in F-score in S6 Fig and S6 Fig. We found also that the fraction of (wrongly) directed edges is close to zero at the predicted effective number of samples, $N_{\text{eff}}^{*}$, providing that it is not too small, *i.e.* $N_{\text{eff}}^{*}>300$.

These results demonstrate that the theoretical estimate of $N_{\text{eff}}^{*}$, Eq 15, yields the best compromise between over-fitting and under-fitting graphical models given the finite and partially correlated available datasets. They underline also miic accuracy to discard spurious causality in observational data, even from relatively small effective numbers of independent samples, *i.e.* $N_{\text{eff}}^{*}>300$ in S6 Fig and S6 Fig.

## Supporting information

S1 File Supplementary text. Contents: **1**. Information-theoretic approach to network reconstruction; **1.1**. Signature of causality *versus* indirect contributions to information in graphs; **1.2**. Finite size effect and most likely contributor score. **2**. Algorithmic pipeline of the information-theoretic approach miic; **2.1**. Algorithm 1: Learning skeleton taking into account latent variables; **2.2**. Algorithm 2: Confidence estimation and sign of retained edges; **2.3**. Algorithm 3: Probabilistic orientation and propagation of remaining edges. **3**. Algorithmic implementation and tools; **3.1**. miic R-package; **3.2**. miic and FCI executables. **4**. References for Supplementary Text.
(PDF)

S1 Fig Real-life Alarm network with hidden latent variables. [37 nodes, 46 links, 509 parameters, Average degree 2.49, Maximum in-degree 4]. Precision, Recall, F-score and computing time for PAG skeletons (dashed lines) and PAGs including orientations (solid lines). The results are given for the miic algorithm (warm colors) compared to the RFCI algorithm [10] (cold colors) for 0, 2, 4 and 6 latent variables out of the 37 nodes. Computation times in log scale show a linear scaling in the limit of large datasets, *τ*_cpu_ ∼ *N*^0.9^, for the miic algorithm, and a stronger nonlinear increase, *τ*_cpu_ ∼ *N*^1.5^, with the RFCI algorithm.
(TIFF)

S2 Fig Real-life Insurance network with hidden latent variables. [27 nodes, 52 links, 984 parameters, Average degree 3.85, Maximum in-degree 3]. Precision, Recall, F-score and computing time for PAG skeletons (dashed lines) and PAGs including orientations (solid lines). The results are given for the miic algorithm (warm colors) compared to the RFCI algorithm [10] (cold colors) for 0, 1, 2, and 4 latent variables out of the 27 nodes. Computation times in log scale show a linear scaling in the limit of large datasets, *τ*_cpu_ ∼ *N*^1.0^, for the miic algorithm, and a stronger nonlinear increase, *τ*_cpu_ ∼ *N*^1.7^, with the RFCI algorithm.
(TIFF)

S3 Fig Real-life Barley network with hidden latent variables. [48 nodes, 84 links, 114,005 parameters, Average degree 3.5, Maximum in-degree 4]. Precision, Recall, F-score and computing time for PAG skeletons (dashed lines) and PAGs including orientations (solid lines). The results are given for the miic algorithm (warm colors) compared to the RFCI algorithm [10] (cold colors) for 0, 2, 4 and 7 latent variables out of the 48 nodes. Computation times in log scale show a nearly linear scaling in the limit of large datasets, *τ*_cpu_ ∼ *N*^1.1^, for the miic algorithm, and a stronger nonlinear increase, *τ*_cpu_ ∼ *N*^2.3^, with the RFCI algorithm.
(TIFF)

S4 Fig Reconstruction of Fig 1C network from simulated data. miic and RFCI [9, 10]*versus* 3off2 [19] and PC [7, 8, 25] reconstructions of Fig 1C network are performed from simulated data generated with Tetrad V, *N* = 10–50,000 samples. Precision, Recall and Fscore are given for skeleton (dashed lines) and PAG including orientations (solid lines).
(TIFF)

S5 Fig Random benchmark networks of increasing size. miic reconstruction of random networks of increasing size (*P* = 10–500 nodes) and fixed average degree 3 from *N* = 1,000 samples generated with Tetrad V. The average CPU time exhibits an optimal quadratic complexity in terms of network size, *τ*_cpu_ ∼ *P*^2^ (solid bar), with only a small time increase when considering latent variables (orange) as compared to excluding them (red).
(TIFF)

S6 FigAlarm-like undirected network.Precision, Recall, F-score, percentage of (wrongly) directed edges and decay of the autocorrelation function with lag between successive samples for *N* = 1,000 to 300,000 consecutive partially correlated samples (with predicted effective number of independent samples in brackets). Vertical dashed lines correspond to the predicted effective number of independent samples $N_{\text{eff}}^{*}/N≃0.068$, see Materials and methods.(TIFF)

S7 FigInsurance-like undirected network.Precision, Recall, F-score, percentage of (wrongly) directed edges and decay of the autocorrelation function with lag between successive samples for *N* = 1,000 to 300,000 consecutive partially correlated samples (with predicted effective number of independent samples in brackets). Vertical dashed lines correspond to the predicted effective number of independent samples $N_{\text{eff}}^{*}/N≃0.069$, see Materials and methods.(TIFF)

S8 Fig Edge confidence filtering on real-life Alarm network. [37 nodes, 46 links, 509 parameters, Average degree 2.49, Maximum in-degree 4]. Precision, Recall, F-score and computing time for network skeleton (dashed lines) and oriented network CPDAG (solid lines) for a decreasing edge-specific confidence filtering, *C*_*XY*_ = 1 (no filtering) 0.01, 0.001 and 0.0001. For sample size >100, confidence filtering of individual edges improves the precision (at the expense of recall) not only for the skeleton (dashed lines), as expected, but also for the oriented networks (solid lines). In addition, limited filtering, *i.e.* keeping edges with *C*_*XY*_ < 10^−3^−10^−2^, tends to yield equivalent F-scores as unfiltered benchmark reconstructions.
(TIFF)

S9 Fig Edge confidence filtering on real-life Insurance network. [27 nodes, 52 links, 984 parameters, Average degree 3.85, Maximum in-degree 3]. Precision, Recall, F-score and computing time for network skeleton (dashed lines) and oriented network CPDAG (solid lines) for a decreasing edge-specific confidence filtering, *C*_*XY*_ = 1 (no filtering) 0.01, 0.001 and 0.0001. For sample size >100, confidence filtering of individual edges improves the precision (at the expense of recall) not only for the skeleton (dashed lines), as expected, but also for the oriented networks (solid lines). In addition, limited filtering, *i.e.* keeping edges with *C*_*XY*_ < 10^−3^−10^−2^, tends to yield equivalent F-scores as unfiltered benchmark reconstructions.
(TIFF)

S10 Fig Gene expression distribution in 3,934 single cells from mouse embryos. Expression data on the 33 TFs are obtained from [24]. Percentage of samples with expressed genes (red) and non-expressed genes (gray).
(TIFF)

S11 Fig Unfiltered network skeleton for hematopoiesis differentiation data. Hematopoietic / endothelial gene expression data in 3,934 single cells from mouse embryos [24]. 7 out of 82 edges (8.5%) with *C*_*XY*_ > 10^−3^ have been filtered in Fig 2D (blue edges correspond to anti-correlations).
(TIFF)

S12 Fig Expression alterations in 807 samples of breast tumor data from COSMIC database [34]. Percentage of samples with normalized over-expression (red), normalized under-expression (blue) and unchanged normalized expression (gray) for each gene based on COSMIC.
(TIFF)

S13 Fig Mutations in 807 samples of breast tumor data from COSMIC database [34]. Percentage of mutated samples (red) for each gene.
(TIFF)

S14 Fig Unfiltered network skeleton for breast tumor ploidy-mutation- expression data from COSMIC database [34]. Due to the limited numbers of samples (N = 807) and recurrent gene mutants (Figure -figure supplement 2), most gene mutations (yellow) are not confidently linked to any altered expression levels (green) and have been filtered in the high confidence network Fig 3D (*C*_*XY*_ < 10^−3^), with the notable exceptions of *TP53* and *RB1* mutations, which have a significant impact on gene expressions, Fig 3D, see main text (blue edges correspond to anti-correlations).
(TIFF)

S15 Fig Unfiltered network skeleton for ohnolog retention data in human. Genomic data for the 20,415 human coding genes is provided in S1 Data. The only edge with confidence ratio *C*_*XY*_ > 10^−3^ is RecDominance − ProteinComplex with *C*_*XY*_ = 0.25 (blue edges correspond to anti-correlations).
(TIFF)

S1 Software Software and tools. miic software is provided in two formats: an R-package to be used in the R environment, and miic and FCI executables, which were used for all benchmarks included in the paper.
(ZIP)

S1 Data Dataset of human genomic properties. This dataset contains all genomic data for the 20,415 human genes analyzed in Fig 4.
(XLS)

S1 Table Effect of BIC/MDL *versus* NML criteria in applications. Choosing the BIC/MDL instead of NML criterion in the three genetic network applications, Figs 2, 3 & 4, leads to somewhat sparser reconstituted networks including 82% to 100% of initial edges, yet no additional edges (*i.e.* consistent with a lower recall), and 66% to 75% conserved edge orientations (*i.e.* identical −, →, ← and ↔ edges).
(XLS)
