# Supplementary material for: Learning causal networks with latent variables from multivariate information in genomic data
Source: PLoS Comput Biol. 2017 Oct 2;13(10):e1005662. doi: 10.1371/journal.pcbi.1005662 (PMC5685645; doi:10.1371/journal.pcbi.1005662)
Supplement: S1 File — Contents: 1. Information-theoretic approach to network reconstruction; 1.1. Signature of causality versus indirect contributions to information in graphs; 1.2. Finite size effect and most likely contributor score. 2. Algorithmic pipeline of the information-theoretic approach miic; 2.1. Algorithm 1: Learning skeleton taking into account latent variables; 2.2. Algorithm 2: Confidence estimation and sign of retained edges; 2.3. Algorithm 3: Probabilistic orientation and propagation of remaining edges. 3. Algorithmic implementation and tools; 3.1. miic R-package; 3.2. miic and FCI executables. 4. References for Supplementary Text. (PDF) [file pcbi.1005662.s001.pdf]

Supplementary Text

on manuscript

Learning causal networks with latent variables from multivariate information in genomic data

Louis Verny[1,2◕], Nadir Sella[1,2◕], Séverine Affeldt[1,2◕¤a], Param Priya Singh[1,2¤b] & Hervé Isambert[1,2*]

[1]Institut Curie, PSL Research University, CNRS, UMR168, 26 rue dUlm, 75005 Paris, France
[2]Sorbonne Universités, UPMC Univ Paris 06, 4, Place Jussieu, 75005 Paris, France

¤a Current address: LIPADE, University of Paris Descartes, 45 rue des Saints Pères, 75006 Paris, France
¤b Current address: Department of Genetics, Stanford University, Palo Alto, USA

◕These authors contributed equally to this work.
*To whom correspondence should be addressed; E-mail: herve.isambert@curie.fr

Contents

1 Information-theoretic approach to network reconstruction **2**
1.1 Signature of causality *versus* indirect contributions to information in graphs 2
1.2 Finite size effect and most likely contributor score 3

2 Algorithmic pipeline of the information-theoretic approach `miic` **6**
2.1 Algorithm 1: Learning skeleton taking into account latent variables 6
2.2 Algorithm 2: Confidence estimation and sign of retained edges 8
2.3 Algorithm 3: Probabilistic orientation and propagation of remaining edges 9

3 Algorithmic implementation and tools **11**
3.1 `miic` R-package 11
3.2 `miic` and `FCI` executables 12

4 References for Supplementary Text **16**

1 Information-theoretic approach to network reconstruction

1.1 Signature of causality *versus* indirect contributions to information in graphs

We first discuss the rationale of the information-theoretic method to learn ancestral graphs with the assumption that an infinite amount of data is available with a distribution $P(\boldsymbol{X})$ faithful to an underlying graph model $\mathcal{G}$, before discussing in the next section the necessary corrections needed, in practice, to account for the finite size of the dataset.

We will thus assume that the measured distribution $P(\boldsymbol{X})$ is stable or faithful to the underlying graph model $\mathcal{G}$, implying that each structural independency under m-separation criterion [1] (*i.e.* each excluded edge XY in $\mathcal{G}$) corresponds to a vanishing conditional mutual information as,

$$\begin{aligned}(X \perp_m Y|\{A_i\})_G &\iff (X \perp\!\!\!\perp Y|\{A_i\})_P \\ &\iff I(X;Y|\{A_i\}) = 0\end{aligned} \tag{S1}$$

Theorem 1 [Signature of causality *vs* indirect contributions in $\mathcal{G}$, Affeldt & Isambert 2015 [2]] *Given some data with a distribution $P(\boldsymbol{X})$ faithful to a graph $\mathcal{G}$,*

- i) *[Signature of causality] If $\exists X,Y,Z \in \boldsymbol{V}$ and $\{A_i\} \subseteq \boldsymbol{V}\backslash\{X,Y,Z\}$ s.t. $I(X;Y|\{A_i\}) = 0$ and $I(X;Y;Z|\{A_i\}) < 0$, then $\mathcal{G}$ is necessarily causal, i.e. it has at least one v-structure.*
- ii) *[Indirect contribution] $\forall X,Y,Z \in \boldsymbol{V}$ and $\forall\{A_i\} \subseteq \boldsymbol{V}\backslash\{X,Y,Z\}$ s.t. $I(X;Y;Z|\{A_i\}) > 0$, then $I(X;Y|\{A_i\}) = I(X;Y;Z|\{A_i\}) + I(X;Y|Z,\{A_i\}) > 0$ and $I(X;Y;Z|\{A_i\}) > 0$ can be seen as the positive contribution to the remaining conditional mutual information $I(X;Y|\{A_i\}) > 0$ (and equivalently to $I(X;Z|\{A_i\}) > 0$ and $I(Y;Z|\{A_i\}) > 0$ by symmetry of $I(X;Y;Z|\{A_i\})$).*

Sketch of proof [The full proof is given in [2]: Theorem 4 and Corollary 2] *i)* is proven via its contrapositive showing that non-causal graphs with structural independence $I(X;Y|\{A_i\}) = 0$ have necessarily vanishing conditional three-point information terms, $I(X;Y;Z|\{A_i\}) = 0, \forall Z \in \boldsymbol{V} \setminus (\{X,Y\} \cup \{A_i\})$. Moreover, since $I(X;Y;Z|\{A_i\}) \leqslant 0$ whenever $I(X;Y|\{A_i\}) = 0$ for any graph in general (from Eq. 5 in main text with $A_n = Z$), it means that $I(X;Y;Z|\{A_i\}) < 0$ indeed implies a necessary causal graph. *ii)* is simply Eq. 5 in main text with $A_n = Z$ as well. □

Theorem 1 *i)*, which characterizes the signature of causality in observational data, will be used to orient v-structures, once Theorem 1 *ii)* has been used to learn structural independences by collecting one-by-one the significant contributors $\{A_i\}$ and partitioning iteratively mutual information terms into positive contributions from indirect paths as,

$$\begin{aligned}I(X;Y) &= I(X;Y;A_1) + I(X;Y|A_1) \\ &= I(X;Y;A_1) + I(X;Y;A_2|A_1) + I(X;Y|A_1,A_2) \\ &= I(X;Y;A_1) + I(X;Y;A_2|A_1) + \ldots \\ &\quad \ldots + I(X;Y;A_n|\{A_i\}_{n-1}) + I(X;Y|\{A_i\}_n)\end{aligned} \tag{S2}$$

with $I(X;Y;A_k|\{A_i\}_{k-1}) > 0$ for all k. Hence, conditional independence, $I(X;Y|\{A_i\}_n) = 0$, is eventually retrieved (if it holds) after subtracting successive significant positive three-point conditional information from the original two-point conditional information [2, 3] as,

$$I(X;Y|\{A_i\}_n) \quad = \quad I(X;Y) - I(X;Y;A_1) - \ldots - I(X;Y;A_n|\{A_i\}_{n-1}) \qquad \text{(S3)}$$

The robustness of the approach hinges on picking the most likely contributors first to avoid a later accumulation of incorrect contributors in an attempt to compensate for early errors. Choosing the most likely contributors requires, however, to take into account the finite size of the dataset as detailed in the next section.

1.2 Finite size effect and most likely contributor score

This section, adapted from [2], addresses finite size corrections to multivariate information and introduce a heuristic score to collect the most likely contributors $\{A_i\}_n$ in Eq. S3.

Given N independent samples from some available data $\mathcal{D}$, the Maximum Likelihood, $\mathcal{L}_{\mathcal{D}|\mathcal{G}}$, that they might have been generated by the graphical model $\mathcal{G}$, is given by [4],

$$\mathcal{L}_{\mathcal{D}|\mathcal{G}} = \frac{e^{-NH(p,q)}}{Z_{\mathcal{D},\mathcal{G}}} = \frac{e^{N\sum_{\boldsymbol{x}} p(\boldsymbol{x}) \log q(\boldsymbol{x})}}{Z_{\mathcal{D},\mathcal{G}}} \qquad \text{(S4)}$$

where $H(p,q) = -\sum_{\boldsymbol{x}} p(\boldsymbol{x}) \log q(\boldsymbol{x})$ is the cross entropy between the empirical probability distribution $p(\boldsymbol{x})$ of the data $\mathcal{D}$ and the theoretical probability distribution $q(\boldsymbol{x})$ of the model $\mathcal{G}$, and $H(p) = -\sum_{\boldsymbol{x}} p(\boldsymbol{x}) \log p(\boldsymbol{x})$ is the entropy of the data and $Z_{\mathcal{D},\mathcal{G}}$ a data- and model-dependent factor ensuring proper normalization condition. The structural constraints of the model $\mathcal{G}$ are included in the factorization form of the theoretical probability distribution, $q(\boldsymbol{x})$.

In particular, the conditional mutual information, $I(X;Y|\{A_i\})$, for structural independence, Eq. S3, cannot be exactly zero, given a finite dataset of N independent samples, and has to be compared to a finite threshold, $I(X;Y|\{A_i\}) < k_{X;Y|\{A_i\}}/N$, where $k_{X;Y|\{A_i\}} > 0$ is related to the likelihood normalization ratio between graphs including or excluding edge XY with separation set $\{A_i\}$ [2],

$$\frac{\mathcal{L}_{\mathcal{D}|\mathcal{G}_{\backslash XY|\{A_i\}}}}{\mathcal{L}_{\mathcal{D}|\mathcal{G}}} \quad = \quad \frac{e^{-NI(X;Y|\{A_i\})}}{Z_{\mathcal{D},\mathcal{G}_{\backslash XY|\{A_i\}}}/Z_{\mathcal{D},\mathcal{G}}} = e^{-NI(X;Y|\{A_i\})+k_{X;Y|\{A_i\}}} \qquad \text{(S5)}$$

$$k_{X;Y|\{A_i\}} \quad = \quad \log\left(Z_{\mathcal{D},\mathcal{G}}/Z_{\mathcal{D},\mathcal{G}_{\backslash XY|\{A_i\}}}\right) \qquad \text{(S6)}$$

where $k_{X;Y|\{A_i\}}$ tends to limit the complexity of the models by favoring fewer edges. A common complexity criterion in model selection is the Bayesian Information Criterion (BIC) or Minimum Description Length (MDL) criterion [5,6], which is simply related to the maximum likelihood normalization constant reached in the asymptotic limit of a large dataset $N \to \infty$ (Laplace approximation). However, this limit distribution is only reached for very large datasets in practice. Alternatively, the normalization of the maximum likelihood can also be done over all possible datasets including the same number of samples to yield a (universal) Normalized Maximum Likelihood (NML) criterion [7, 8] and its decomposable

version [9, 10]. All application results presented in this paper are obtained with the XY-symmetric decomposable NML criterion introduced in [3], which was shown to yield significantly better results than BIC/MDL criterion on benchmark networks.

Thus, finite size effects in graphical model comparison can be included by redefining two-point and three-point conditional multivariate information as,

$$I'(X;Y|\{A_i\}) = I(X;Y|\{A_i\}) - \frac{k_{X;Y|\{A_i\}}}{N} \quad \text{(S7)}$$

$$I'(X;Y;Z|\{A_i\}) = I(X;Y;Z|\{A_i\}) - \frac{k_{X;Y;Z|\{A_i\}}}{N} \quad \text{(S8)}$$

where conditional three-point information including finite size corrections, $I'(X;Y;Z|\{A_i\})$, and their associated complexity terms, $k_{X;Y;Z|\{A_i\}}$, are defined with respect to two-point information including finite size corrections and their associated complexity terms, using the same Eq. 5 in main text with $A_n = Z$,

$$I'(X;Y;Z|\{A_i\}) = I'(X;Y|\{A_i\}) - I'(X;Y|\{A_i\}, Z) \quad \text{(S9)}$$

$$k_{X;Y;Z|\{A_i\}} = k_{X;Y|\{A_i\}} - k_{X;Y|\{A_i\},Z} \quad \text{(S10)}$$

Hence, Eq. S3 including finite size corrections becomes,

$$I'(X;Y|\{A_i\}_n) = I'(X;Y) - I'(X;Y;A_1) - \ldots - I'(X;Y;A_n|\{A_i\}_{n-1}) \quad \text{(S11)}$$

where the conditional two-point and tree-point multivariate information are related to the following maximum likelihood ratios, using Eq. S6,

$$\frac{\mathcal{L}_{\mathcal{D}|\mathcal{G}_{\backslash XY|\{A_i\}}}}{\mathcal{L}_{\mathcal{D}|\mathcal{G}}} = e^{-NI'(X;Y|\{A_i\})} \quad \text{(S12)}$$

$$\frac{\mathcal{L}_{\mathcal{D}|\mathcal{G}_{\backslash XY|\{A_i\},Z}}}{\mathcal{L}_{\mathcal{D}|\mathcal{G}_{\backslash XY|\{A_i\}}}} = e^{NI'(X;Y;Z|\{A_i\})} \quad \text{(S13)}$$

with conditional independence including finite size effect corresponding to $I'(X;Y|\{A_i\}) \leqslant 0$.

Hence, learning, iteratively, the most likely edge to be removed XY and its corresponding separation set $\{A_i\}$ will imply to simultaneously minimize two-point information (Eq. S12) while maximizing three-point information (Eq. S13). In fact, the sign and magnitude of conditional three-point information included finite size corrections, $I'(X;Y;Z|\{A_i\})$, determine the probability that Z should be included in or excluded from the sepset candidate $\{A_i\}$ as:

- If $I'(X;Y;Z|\{A_i\}) > 0$, Z is more likely to be included in $\{A_i\}$ with probability,

$$P_{\mathsf{nv}}(X;Y;Z|\{A_i\}) = \frac{\mathcal{L}_{\mathcal{D}|\mathcal{G}_{\backslash XY|\{A_i\},Z}}}{\mathcal{L}_{\mathcal{D}|\mathcal{G}_{\backslash XY|\{A_i\}}} + \mathcal{L}_{\mathcal{D}|\mathcal{G}_{\backslash XY|\{A_i\},Z}}} = \frac{1}{1 + e^{-NI'(X;Y;Z|\{A_i\})}} \quad \text{(S14)}$$

- If $I'(X;Y;Z|\{A_i\}) < 0$, Z is more likely to be excluded from $\{A_i\}$, suggesting obligatory causal relationships in the form of a v-structure between X, Y, Z with probability,

$$P_{\mathsf{v}}(X;Y;Z|\{A_i\}) \;=\; 1 - P_{\mathsf{nv}}(X;Y;Z|\{A_i\}) = \frac{1}{1+e^{NI'(X;Y;Z|\{A_i\})}} \tag{S15}$$

But, in the case $I'(X;Y;Z|\{A_i\}) > 0$, Eq. S13 can also be interpreted as quantifying the likelihood increase that the edge XY should be removed from the model by extending the candidate sepset from $\{A_i\}$ to $\{A_i\}+Z$, *i.e.* $\mathcal{L}_{\mathcal{D}|\mathcal{G}_{\backslash XY|\{A_i\},Z}} = \mathcal{L}_{\mathcal{D}|\mathcal{G}_{\backslash XY|\{A_i\}}} \times \exp(NI'(X;Y;Z|\{A_i\})) > \mathcal{L}_{\mathcal{D}|\mathcal{G}_{\backslash XY|\{A_i\}}}$, as $\exp(NI'(X;Y;Z|\{A_i\})) > 1$. Yet, as the three-point information, $I'(X;Y;Z|\{A_i\})$, is actually symmetric with respect to the variables, X, Y and Z, the factor $\exp(NI'(X;Y;Z|\{A_i\}))$ provides in fact the same likelihood increase for the removal of the three edges XY, XZ and ZY, conditioned on the same initial set of nodes $\{A_i\}$, namely,

$$\frac{\mathcal{L}_{\mathcal{D}|\mathcal{G}_{\backslash XY|\{A_i\},Z}}}{\mathcal{L}_{\mathcal{D}|\mathcal{G}_{\backslash XY|\{A_i\}}}} \;=\; \frac{\mathcal{L}_{\mathcal{D}|\mathcal{G}_{\backslash XZ|\{A_i\},y}}}{\mathcal{L}_{\mathcal{D}|\mathcal{G}_{\backslash XZ|\{A_i\}}}} = \frac{\mathcal{L}_{\mathcal{D}|\mathcal{G}_{\backslash ZY|\{A_i\},x}}}{\mathcal{L}_{\mathcal{D}|\mathcal{G}_{\backslash ZY|\{A_i\}}}} = e^{NI'(X;Y;Z|\{A_i\})} \tag{S16}$$

However, despite this symmetry of three-point information, $I'(X;Y;Z|\{A_i\})$, the likelihoods that the edges XY, XZ and ZY should be removed are not the same, as they depend on different 2-point information, $I'(X;Y|\{A_i\})$, $I'(X;Z|\{A_i\})$ and $I'(Z;Y|\{A_i\})$, Eq. S12. In particular, the likelihood ratio between the removals of the alternative edges XY and XZ is given by,

$$\frac{\mathcal{L}_{\mathcal{D}|\mathcal{G}_{\backslash XY|\{A_i\},Z}}}{\mathcal{L}_{\mathcal{D}|\mathcal{G}_{\backslash XZ|\{A_i\},Y}}} \;=\; \frac{\mathcal{L}_{\mathcal{D}|\mathcal{G}_{\backslash XY|\{A_i\}}}}{\mathcal{L}_{\mathcal{D}|\mathcal{G}_{\backslash XZ|\{A_i\}}}} = \frac{e^{-NI'(X;Y|\{A_i\})}}{e^{-NI'(X;Z|\{A_i\})}} \tag{S17}$$

and similarly between edges XY and ZY.

Hence, for XY to be the most likely edge to be removed conditioned on the sepset $\{A_i\}+Z$, not only Z should contribute through $I'(X;Y;Z|\{A_i\})>0$ with probability $P_{\mathsf{nv}}(X;Y;Z|\{A_i\})$ (Eq. S14), but XY must also correspond to the 'weakest' edge of XY, XZ and ZY conditioned on $\{A_i\}$, as given by the lowest conditioned 2-point information, Eq. S17. Note that removing the edge XY with the lowest conditional 2-point information is consistent, as expected, with the Data Processing Inequality, $I(X;Y|\{A_i\}) \leqslant \min(I(X;Z|\{A_i\}), I(Z;Y|\{A_i\}))$, in the limit of large datasets. However, quite frequently, XZ or ZY might also have low conditional 2-point information, so that the edge removal associated with the symmetric contribution $I(X;Y;Z|\{A_i\})$ will only be consistent with the Data Processing Inequality (DPI) with probability,

$$\begin{aligned} P_{\mathsf{dpi}}(XY;Z|\{A_i\}) \;&=\; \frac{\mathcal{L}_{\mathcal{D}|\mathcal{G}_{\backslash XY|\{A_i\}}}}{\mathcal{L}_{\mathcal{D}|\mathcal{G}_{\backslash XY|\{A_i\}}} + \mathcal{L}_{\mathcal{D}|\mathcal{G}_{\backslash XZ|\{A_i\}}} + \mathcal{L}_{\mathcal{D}|\mathcal{G}_{\backslash ZY|\{A_i\}}}} \\ &=\; \frac{1}{1 + \frac{e^{-NI'(X;Z|\{A_i\})}}{e^{-NI'(X;Y|\{A_i\})}} + \frac{e^{-NI'(Z;Y|\{A_i\})}}{e^{-NI'(X;Y|\{A_i\})}}} \end{aligned} \tag{S18}$$

In practice, taking into account this DPI-consistency probability $P_{\mathsf{dpi}}(XY;Z|\{A_i\})$, as detailed below, significantly improves the results obtained by relying solely on the 'non-v-structure' probability

$P_{\mathsf{nv}}(X;Y;Z|\{A_i\})$. Conversely, the DPI-consistency probability $P_{\mathsf{dpi}}(XY;Z|\{A_i\})$ is not sufficient on its own to uncover causal relationships between variables, which require to compute three-point information $I(X;Y;Z|\{A_i\})$ and the probability $P_{\mathsf{nv}}(X;Y;Z|\{A_i\})$ (see Proposition 1 and Proposition 2, below).

To optimize the likelihood that the edge XY can be accounted for by the additional contribution of Z conditioned on previously selected $\{A_i\}$, we propose to combine the maximum of three-point information (Eq. S14) and the minimum of 2-point information (Eq. S18) by defining the score $S_{\mathsf{lb}}(Z;XY|\{A_i\})$ as the lower bound of $P_{\mathsf{nv}}(X;Y;Z|\{A_i\})$ and $P_{\mathsf{dpi}}(XY;Z|\{A_i\})$, since both conditions need to be fulfilled to warrant that edge XY is likely to be absent from the model $\mathcal{G}$,

$$S_{\mathsf{lb}}(Z;XY|\{A_i\}) = \min\left[P_{\mathsf{nv}}(X;Y;Z|\{A_i\}), P_{\mathsf{dpi}}(XY;Z|\{A_i\})\right] \tag{S19}$$

Hence, the pair of nodes XY with the most likely contribution from a third node Z and likely to be absent from the model can be ordered according to their rank $R(XY;Z|\{A_i\})$ defined as,

$$R(XY;Z|\{A_i\}) = \max_Z \left(S_{\mathsf{lb}}(Z;XY|\{A_i\})\right) \tag{S20}$$

Then, Z can be iteratively added to the set of contributing nodes (*i.e.* $\{A_i\} \leftarrow \{A_i\} + Z$) of the top edge $XY = \text{argmax}_{XY} R(XY;Z|\{A_i\})$ to progressively recover the most significant indirect contributions to all pairwise mutual information in a causal graph.

2 Algorithmic pipeline of the information-theoretic approach `miic`

The implementation of the information-theoretical approach `miic` proceeds in three steps corresponding to the following algorithmic pipeline:

- Algorithm 1: Learning skeleton taking into account latent variables
- Algorithm 2: Confidence estimation and sign of retained edges
- Algorithm 3: Probabilistic orientation and propagation of remaining edges

`miic` is implemented in an R-package and freely available under a General Public License (Supplementary Software).

2.1 Algorithm 1: Learning skeleton taking into account latent variables

Using the heuristic score, Eq. S20, to implement the successive subtractions of three-point conditional information terms in Eq. S11, yields Algorithm 1

Algorithm 1: Skeleton reconstruction in the presence of latent variables

In: observational data of finite size N, complexity criterion NML (or MDL)
Out: skeleton of ancestral graph $\mathcal{G}$

Initiation

Start with complete undirected graph

forall the *edges* XY **do**
    if $I'(X;Y) < 0$ **then**
        XY **edge is** non-essential and **removed**
        separation set of XY: $\mathrm{Sep}_{XY} = \emptyset$
    else
        find the **most contributing node** Z and **compute its rank**, $R(XY;Z|\emptyset)$
        (*Z can be restricted to neighbors of X and Y if latent variables are excluded*)
    end
end

Iteration

while $\exists$ XY *edge with* $R(XY;Z|\{A_i\}) > 1/2$ **do**
    for *edge* XY *with highest rank* $R(XY;Z|\{A_i\})$ **do**
        expand contributing set $\{A_i\} \leftarrow \{A_i\} + Z$
        if $I'(X;Y|\{A_i\}) < 0$ **then**
            XY **edge is** non-essential and **removed**
            separation set of XY: $\mathrm{Sep}_{XY} = \{A_i\}$
        else
            find the **next most contributing node** Z and **compute rank**, $R(XY;Z|\{A_i\})$
            (*Z can be restricted to neighbors of X and Y if latent variables are excluded*)
        end
        update highest rank edge
    end
end

2.2 Algorithm 2: Confidence estimation and sign of retained edges

Once a first skeleton has been obtained using Algorithm 1, the confidence on each retained edge can be estimated through an edge specific confidence ratio C_{XY} based on the probability P_{XY} to remove a directed edge $X \to Y$ from the graph $\mathcal{G}$, as defined by Eq. S12,

$$P_{XY} = \frac{\mathcal{L}_{\mathcal{D}|\mathcal{G}_{\setminus XY|\{A_i\}}}}{\mathcal{L}_{\mathcal{D}|\mathcal{G}}} = e^{-NI'(X;Y|\{A_i\})} \tag{S21}$$

$$C_{XY} = \frac{P_{XY}}{\langle P_{XY}^{\text{rand}} \rangle} \tag{S22}$$

where $\langle P_{XY}^{\text{rand}} \rangle$ is the average of the probability to remove the XY edge after randomly permutating the dataset for each observable. Hence, the lower C_{XY}, the higher the confidence on the XY edge.

In practice, $\langle P_{XY}^{\text{rand}} \rangle$ is not actually evaluated looking for contributors $\{A_i\}$ as done for P_{XY} (since there should be no contributors nor edges after randomization of the data) but just computing $\langle P_{XY}^{\text{rand}} \rangle = \langle e^{-NI'(X^{\text{rand}};Y)} \rangle$, where the X^{rand} variable is assigned randomly permutated values of X across the different samples (randomizing Y or both variables is statistically equivalent). As a result, C_{XY} is slightly overestimated (as ignoring contributors actually underestimates $\langle P_{XY}^{\text{rand}} \rangle$) but can be computed efficiently by averaging over hundreds of permutated values at each vertex. The filtering of retained edges is implemented in Algorithm 2.

Algorithm 2: Filtering retained edges according to an edge specific confidence ratio C_{XY}

In: Skeleton obtained from Algorithm 1, confidence level $C_s < 1$, nb permutations $r_{\max}$
Out: Revised skeleton, after filtering out lower confidence edges with $C_{XY} > C_s$

```
forall the vertices X_i do
    forall the random permutations r < r_max do
        Assign X_i^rand values through random permutation of X_i values
        forall the X_j adjacent of X_i with j > i do
            Compute I'_r(X_i^rand; X_j) ← max(0, I'(X_i^rand; X_j))
        end
    end
    forall the X_j adjacent of X_i with j > i do
        Compute ⟨P^rand_{X_i X_j}⟩ = ⟨e^{-N I'_r(X_i^rand; X_j)}⟩_{r_max}
        Compute C_{X_i X_j} = P_{X_i X_j} / ⟨P^rand_{X_i X_j}⟩ and remove edge X_i X_j, if C_{X_i X_j} > C_s
    end
end
```

In addition, the sign of each retained edge, $X - Y$, is defined by the sign of the partial correlation coefficient, $\rho_{XY \cdot \boldsymbol{A}}$, between X and Y conditioned on its derived contributors $\boldsymbol{A} = \{A_i\}$ in Algorithm 1,

with positive coefficients corresponding to partial correlations and negative coefficients corresponding to partial anti-correlations.

The partial correlation coefficient can be computed in $\mathcal{O}(n^3)$ using matrix inversion of the partial covariance matrix (on $\{X, Y\} \cup \boldsymbol{A}$ variables) or by dynamic programming using the recursive formula, for any $A_\circ \in \boldsymbol{A}$,

$$\rho_{XY\cdot\boldsymbol{A}} = \frac{\rho_{XY\cdot\boldsymbol{A}\backslash\{A_\circ\}} - \rho_{XA_\circ\cdot\boldsymbol{A}\backslash\{A_\circ\}}\rho_{A_\circ Y\cdot\boldsymbol{A}\backslash\{A_\circ\}}}{\sqrt{1-\rho^2_{XA_\circ\cdot\boldsymbol{A}\backslash\{A_\circ\}}}\sqrt{1-\rho^2_{A_\circ Y\cdot\boldsymbol{A}\backslash\{A_\circ\}}}} \tag{S23}$$

Negative partial correlations are represented as blue edges in the predicted network reconstructions, Figures 2-4 and their figure supplements.

2.3 Algorithm 3: Probabilistic orientation and propagation of remaining edges

Given the skeleton obtained from Algorithm 1, possibly filtered through Algorithm 2, based on edge specific confidence ratio, Eqs. S14 and S15 can then be used to establish the following Proposition 1 and Proposition 2 for probabilistic orientation and propagation rules of unshielded triples.

To this end, let us first introduce three different endpoint marks associated to edges in mixed graphs: they are the tail ($-$), the head ($>$) and the unspecified ($\circ$) endpoint marks. In addition, we will use the asterisk symbol ($*$) as a wild card denoting any of the three marks and define orientation probabilities at either one or two (underlined) endmarks using Propositions 1 and 2 below.

Proposition 1 [Robust orientation of v-structures from finite dataset including latent variables]
*Assuming that the underlying graphical model is an ancestral graph $\mathcal{G}$ on $\boldsymbol{V}$,
if $\exists X, Y, Z, \{A_i\} \in V$ s.t. $I'(X;Y;Z|\{A_i\}) < 0$ then,*

i. if X, Y, Z form an unshielded triple, $X \ *\!\!-\!\!\circ\ Z \ \circ\!\!-\!\!*\ Y$ with $X \not- Y$, then it should be oriented as $X *\!\!\rightarrow Z \leftarrow\!\!* Y$, with endmark probabilities at $\underline{Z}$,

$$P^\circ_{X*\!\rightarrow\underline{Z}} = P^\circ_{Y*\!\rightarrow\underline{Z}} = \frac{1+e^{NI'(X;Y;Z|\{A_i\})}}{1+3e^{NI'(X;Y;Z|\{A_i\})}} \tag{S24}$$

ii. similarly, if X, Y, Z form an unshielded triple, with one already known converging arrow into the middle node, $X *\!\!\rightarrow Z \ \circ\!\!-\!\!*\ Y$, with endmark probability at $\underline{Z}$, $P_{X*\!\rightarrow\underline{Z}} > P^\circ_{X*\!\rightarrow\underline{Z}}$, then the second edge should be oriented to form a v-structure, $X *\!\!\rightarrow Z \leftarrow\!\!* Y$, with endmark probability at $\underline{Z}$,

$$P_{Y*\!\rightarrow\underline{Z}} = P_{X*\!\rightarrow\underline{Z}}\left(\frac{1}{1+e^{NI'(X;Y;Z|\{A_i\})}} - \frac{1}{2}\right) + \frac{1}{2} \tag{S25}$$

Proof. The implications (*i.*) and (*ii.*) rely on Eq. S15 to estimate the probability that the two edges form

a v-structure. We start proving (*ii.*) using the probability decomposition formula:

$$\begin{aligned} P_{Y*\!\!\to \underline{Z}} &= P_{X*\!\!\to \underline{Z}} \frac{P_{X*\!\!\to Z \leftarrow\!* Y}}{P_{X*\!\!\to Z \leftarrow\!* Y} + P_{X*\!\!\to Z \to Y}} \\ &\quad + (1 - P_{X*\!\!\to \underline{Z}}) \frac{P_{X \leftarrow Z \leftarrow\!* Y}}{P_{X \leftarrow Z \leftarrow\!* Y} + P_{X \leftarrow Z \to Y}} \\ &= P_{X*\!\!\to \underline{Z}} \left(\frac{1}{1 + e^{NI'(X;Y;Z|\{A_i\})}} - \frac{1}{2} \right) + \frac{1}{2} \end{aligned} \tag{S26}$$

which also leads to (*i.*) if one assumes $P_{X*\!\!\to \underline{Z}} = P_{Y*\!\!\to \underline{Z}}$ by symmetry in absence of prior information on these orientations. □

Following the rationale of constraint-based approaches, it is then possible to 'propagate' further the orientations downstream of v-structures, using Eq. S14 for positive (conditional) three-point information. For simplicity and consistency, we only implement the propagation of orientation based on likelihood ratios, which can be quantified for finite datasets as proposed in the following Proposition 2. Hence, we do not apply the complete propagation rules for ancestral graphs [11], which inforce in particular acyclic constraints, that are necessary to have a complete reconstruction of the Markov equivalent class of the underlying ancestral graph model.

Proposition 2 [Robust propagation of orientations from finite dataset including latent variables] *Assuming that the underlying graphical model is an ancestral graph $\mathcal{G}$ on $\boldsymbol{V}$, $\forall X, Y, Z, \{A_i\} \in V$ s.t. $I'(X;Y;Z|\{A_i\}) > 0$, if X, Y, Z form an unshielded triple with one already known converging orientation, $X *\!\!\to Z \circ\!\!-\!* Y$, with endmark probability at $\underline{Z}$, $P_{X*\!\!\to \underline{Z}} > 1/2$, then this orientation should be 'propagated' to the second edge as $X *\!\!\to Z \to Y$, with endmark probability at $\underline{Z}$ and $\underline{Y}$,*

$$P_{\underline{Z} \to \underline{Y}} = P_{X*\!\!\to \underline{Z}} \left(\frac{1}{1 + e^{-NI'(X;Y;Z|\{A_i\})}} - \frac{1}{2} \right) + \frac{1}{2} \tag{S27}$$

Proof. This results is shown using the probability decomposition formula,

$$\begin{aligned} P_{\underline{Z} \to \underline{Y}} &= P_{X*\!\!\to \underline{Z}} \frac{P_{X*\!\!\to Z \to Y}}{P_{X*\!\!\to Z \leftarrow\!* Y} + P_{X*\!\!\to Z \to Y}} \\ &\quad + (1 - P_{X*\!\!\to Z}) \frac{P_{X \leftarrow Z \to Y}}{P_{X \leftarrow Z \leftarrow\!* Y} + P_{X \leftarrow Z \to Y}} \\ &= P_{X*\!\!\to \underline{Z}} \left(\frac{1}{1 + e^{-NI'(X;Y;Z|\{A_i\})}} - \frac{1}{2} \right) + \frac{1}{2} \end{aligned} \tag{S28}$$

□

Proposition 1 and Proposition 2 lead to the following Algorithm 3 for the orientation of unshielded triples of the graph skeleton obtained from Algorithm 1 with possibly additional edge filtering through Algorithm 2.

Algorithm 3: Probabilistic Orientation / Propagation of edges including latent variables

In: Graph skeleton from Algorithm 1, possibly filtered through Algorithm 2, and corresponding conditional three-point information $I'(X;Y;Z|\{A_i\})$.

Out: Partially oriented causal graph $\mathcal{G}$ with endmark orientation probabilities.

Probabilistic Orientation / Propagation Step including latent variables

sort list of unshielded triples, $\mathcal{L}_c = \{\langle X, Z, Y\rangle_{X \not\to Y}\}$, in decreasing order of their endmark orientation/propagation probabilities initialized at 1/2 and computed from:
- (*i.*) Proposition 1, if $I'(X;Y;Z|\{A_i\}) < 0$, or
- (*ii.*) Proposition 2, if $I'(X;Y;Z|\{A_i\}) > 0$

repeat

Take $\langle X, Z, Y\rangle_{X \not\to Y} \in \mathcal{L}_c$ with highest endmark orient./propa. probability $> 1/2$.

if $I'(X;Y;Z|\{A_i\}) < 0$ **then**

Orient/propagate edge direction(s) to form a **v-structure** $X *\!\!\rightarrow Z \leftarrow\!\!* Y$ with endmark probabilities $P_{X*\!\to \underline{Z}}$ and $P_{Y*\!\to \underline{Z}}$ given by **Proposition 1**.

else

Propagate second edge direction to form a **non-v-structure** $X *\!\!\rightarrow Z \rightarrow Y$ assigning endmark probabilities $P_{\underline{Z}\to\underline{Y}}$ from **Proposition 2**.

end

Apply new orientation(s) and **sort** remaining list of unshielded triples $\mathcal{L}_c \leftarrow \mathcal{L}_c \backslash \langle X, Z, Y\rangle_{X \not\to Y}$ after **updating propagation probabilities**.

until *no additional endmark orient./propa. probability* $> 1/2$;

3 Algorithmic implementation and tools

We provide the `miic` software in two formats, an R-package to be used in the R environment, and executables to be used directly in a terminal.

3.1 `miic` R-package

`miic` R-package contains the full implementation of the method designed for the R environment.

To install `miic`, enter R and install the R package as,

```
install.packages("< path >/miic_0.1.tar.gz", repos=NULL, source=T)
```

The package also provides functions to plot the predicted networks within the R environment and to export the results in graphml format for cytoscape graphical display. See the package documentation for additional details on `miic` R-package.

The R-package contains the three application datasets presented in the main text (Figures 2-4). An example of network reconstruction is detailed below:

```
library(miic)
# load hematopoiesis data frame
data(hematoData)

# execute MIIC (reconstruct graph)
miic.res = miic(inputData = hematoData, latent = TRUE, confidenceShuffle = 100,
confidenceRatio = 0.001)

# plot graph
miic.plot(miic.res)

# write graph to graphml format. Note that to correctly visualize the network we created the miic style for Cytoscape (http://www.cytoscape.org/).
miic.write.cytoscape(g = miic.res, file = "..")
```

3.2 miic and FCI executables

We provide also `miic` and `FCI` executables, which were used for all benchmarks included in the paper.

Directories and scripts

The main folder contains the scripts and source code for the reconstruction of networks from observational data.

The directories are organized as follows:

```
/
|__ common
|   |__ miic.R
|   |__ gmPlot.R
|   |__ gmSummary.R
|__ sharedLib
|__ data
|   |__ some data input/output...
|__ miic
|   |__ all miic scripts and executables
|__ fci
    |__ all fci scripts
```

Package requirements

To launch the miic.R script, R should be installed, along with some packages available in the CRAN repository.

Rpackages getopt, plotrix, igraph, ppcor, bnlearn, pcalg

Calling the inference methods with `miic`

The inference methods can be called through the *miic.R* script.

Overview

main *~/common/miic.R*

lib *~/common/lib/...*

Arguments *(mandatory: *)*

-i * file path of the input dataset[1]

-o * directory path for the output of the inference method[2]

-m inference method (*miic*, *fci*)
default: *miic*

-d steps to perform[3] ('1,2,3,4' or '1,2' or '1,3' *etc...*)
default: '1,2,3,4'

-p parameters for the inference method (see the following subsections). The value expected here is of type character: '$param_1$:$value_1$,$param_2$:$value_2$ *etc...*'

-t file path to the true edges; used during the *summary* step[4]

-l file path to the layout of each vertex; used during the *plot* step[5]

-c if given, edges will be filtered according to their confidence ratio. It needs two parameters, described in the part "Option '-c' for `miic`".

-s if given, this file provides an ordering of each variable categories for the calculation of the sign of the edges. The signs are calculated using Spearman's partial correlation coefficient. The ordering file is automatically generated if not provided by the user and can be edited to properly reorder the variable categories. See an example of stateOrder file in the data directory.

[1]The input dataset should be a tab separated table, with column names but no row names. Missing values should be indicated with *NA*. Each column corresponds to a categorized variable and each row to one sample.

[2]To prevent from overwriting existing results, if the output directory already exists, the skeleton inference step returns a message and stops.

[3](1) skeleton, (2) probabilistic orientation, (3) summary, (4) plot

[4]The true edges file has two space-separated columns. Each line corresponds to one true edge. The orientation is $col1 \rightarrow col2$.

[5]The layout file has three tab separated columns, the first column being optional. Each line corresponds to the (x, y) coordinates of each vertex. The first column can contain the label of the vertex as indicated in the colnames of the input dataset table. The order in which the coordinates are given also corresponds to the order of the colnames of the input dataset table.

A full example to run in 'common' directory:

*Rscript **miic.R -i** ../data/alarm1000samples.txt **-o** ../data/alarmNetwork **-m** miic **-c** csh:100,ccr:0.01 **-p** cpx:nml,efn:1000,lat:yes,prg:yes **-l** ../data/alarmLayout.txt **-s** ../data/alarmStateOrder.tsv*

When calling the available inference methods with *miic.R*, the 'p' option can be used to indicate the chosen parameters. The value expected for this option is of type character: '$param_1$:$value_1$,$param_2$:$value_2$ *etc...*'. The possible $param_i$ and $value_i$ for each method are detailed in the following subsections.

Option '-p' for `miic`

cpx formula used to compute the complexity term ['mdl'[6] or 'nml'[7]]
default: nml (*Ex.: -p '...,cpx:mdl,...'*)

lat should the network be reconstructed under the hypothesis that some variables might not be observed? ['yes' or 'no']
default: no (*Ex.: -p ...,lat:yes,...*)

prg should the network be oriented using the propagation rule? ['yes' or 'no']
default: yes (*Ex.: -p ...,prg:yes,...*)

efn number of uncorrelated samples
default: number of rows of the input dataset (*Ex.: -p ...,efn:1000,...*)

A '-p' example: *-p cpx:mdl,efn:1000*

Option '-p' for `FCI`

aph α significance level for the statistical independence tests[8]
default: 0.01 (*Ex.: -p ...,aph:0.001,...*)

cit type of statistical independence test ['*gaussCItest*', '*dsepTest*', '*disCItest*', '*binCItest*']
default: gaussCItest (*Ex.: -p ...,cit:disCItest,...*)

skm choose the original PC or the order-independant version ['*stable*', '*original*', '*stable.fast*']
default: stable (*Ex.: -p ...,skm:original,...*)

typ which type of fci variant should be used[9] ['*normal*', '*rapid*', '*adaptive*', '*anytime*']
default: normal (*Ex.: -p ...,typ:rapid,...*)

con set the state of the conservative rule ['*TRUE*', '*FALSE*']
default: FALSE (*Ex.: -p ...,con:TRUE,...*)

[6]Minimum Description Length or Bayesian Information Criterion (BIC) [5, 6]
[7]Normalized Maximum Likelihood criterion [3, 7–10]
[8]refer to the documentation of the R package 'pcalg' for details
[9]RFCI corresponds to 'rapid'

maj set the state of the majority rule [‘*TRUE*’, ‘*FALSE*’]
default: FALSE (*Ex.: -p ...,maj:TRUE,...*)

A full ‘-p’ example: *-p aph:0.000001,cit:disCItest,skm:stable,typ:normal,con:FALSE,maj:FALSE*

Option ‘-c’ for `miic`

csh number of random shuffling of the input dataset, in order to get the random mutual information between `miic` inferred edges (*Ex.: -c ...,csh:100,...*)

ccr confidence ratio used as a threshold for filtering the edges. (*Ex.: -c ...,ccr:0.01,...*)

A full ‘-c’ example: *-c csh:100,ccr:0.01*

Viewing inferred networks

The inferred networks can be viewed either in pdf format (automatically generated with igraph http://igraph.org/) or with interactive graphml format for better display using cytoscape (http://www.cytoscape.org/). The files are located in the following directories:

- Unfiltered network: ‘edgesList.miic.summary.plot_confidence.pdf’ (only with the -m miic option), ‘edgesList.miic.summary.plot_pCor.pdf’ and graphml ‘[output name].graphml’ files are located in the output directory set by the -o entry in the command line
- Filtered networks (using -c option with `miic`): pdf and graphml files are in the subdirectory ‘shuffle_[cshValue]/filtered_network_[ccrValue]’, which can be found in the output directory set by the -o entry in the command line. The output folder name in this case is ‘filtered_network_[ccrValue]’.

We recommend the utilization of Cytoscape tool, version 3.1.0 or later is available for Windows, Linux and OsX. Visualizing `miic` networks with Cytoscape requires to go through the following steps:

1. Import the network: File⇒Import⇒Network⇒File, and select the graphml file in the created output directory
2. Import the style: File⇒Import⇒Styles, and select the miic_style.xml file present at the root of the provided source directory
3. Select the loaded style: under the Style panel present in Control Panel select the miic_style

Detailed information on output files

Additional information on output files can be found in the user manual.

4 References for Supplementary Text

[1] Richardson T, Spirtes P. Ancestral graph Markov models. Ann Statist. 2002;30(4):962–1030. doi:10.1214/aos/1031689015.

[2] Affeldt S, Isambert H. Robust reconstruction of causal graphical models based on conditional 2-point and 3-point information. In: Proceedings of the Thirty-First Conference on Uncertainty in Artificial Intelligence, UAI 2015; 2015. p. 42–51.

[3] Affeldt S, Verny L, Isambert H. 3off2: A network reconstruction algorithm based on 2-point and 3-point information statistics. BMC Bioinformatics. 2016;17(S2).

[4] Sanov IN. On the probability of large deviations of random variables. Mat Sbornik. 1957;42:11–44.

[5] Rissanen J. Modeling by shortest data description. Automatica. 1978;vol. 14:465–471.

[6] Hansen MH, Yu B. Model Selection and the Principle of Minimum Description Length. J Am Stat Assoc. 2001;96:746–774.

[7] Shtarkov YM. Universal sequential coding of single messages. Problems of Information Transmission. 1987;23(3):3–17.

[8] Rissanen J, Tabus I. Kolmogorovs Structure function in MDL theory and lossy data compression. In: Adv. Min. Descrip. Length Theory Appl. MIT Press; 2005. p. 245–264.

[9] Kontkanen P, Myllymäki P. A linear-time algorithm for computing the multinomial stochastic complexity. Inf Process Lett. 2007;103(6):227–233.

[10] Roos T, Silander T, Kontkanen P, Myllymäki P. Bayesian network structure learning using factorized NML universal models. In: Proc. 2008 Information Theory and Applications Workshop (ITA-2008). IEEE Press; 2008.

[11] Zhang J. On the completeness of orientation rules for causal discovery in the presence of latent confounders and selection bias. Artif Intell. 2008;172(16-17):1873–1896. doi:10.1016/j.artint.2008.08.001.
