# Supplementary figures and images for: Learning causal networks with latent variables from multivariate information in genomic data
Source: PLoS Comput Biol. 2017 Oct 2;13(10):e1005662. doi: 10.1371/journal.pcbi.1005662 (PMC5685645; doi:10.1371/journal.pcbi.1005662)

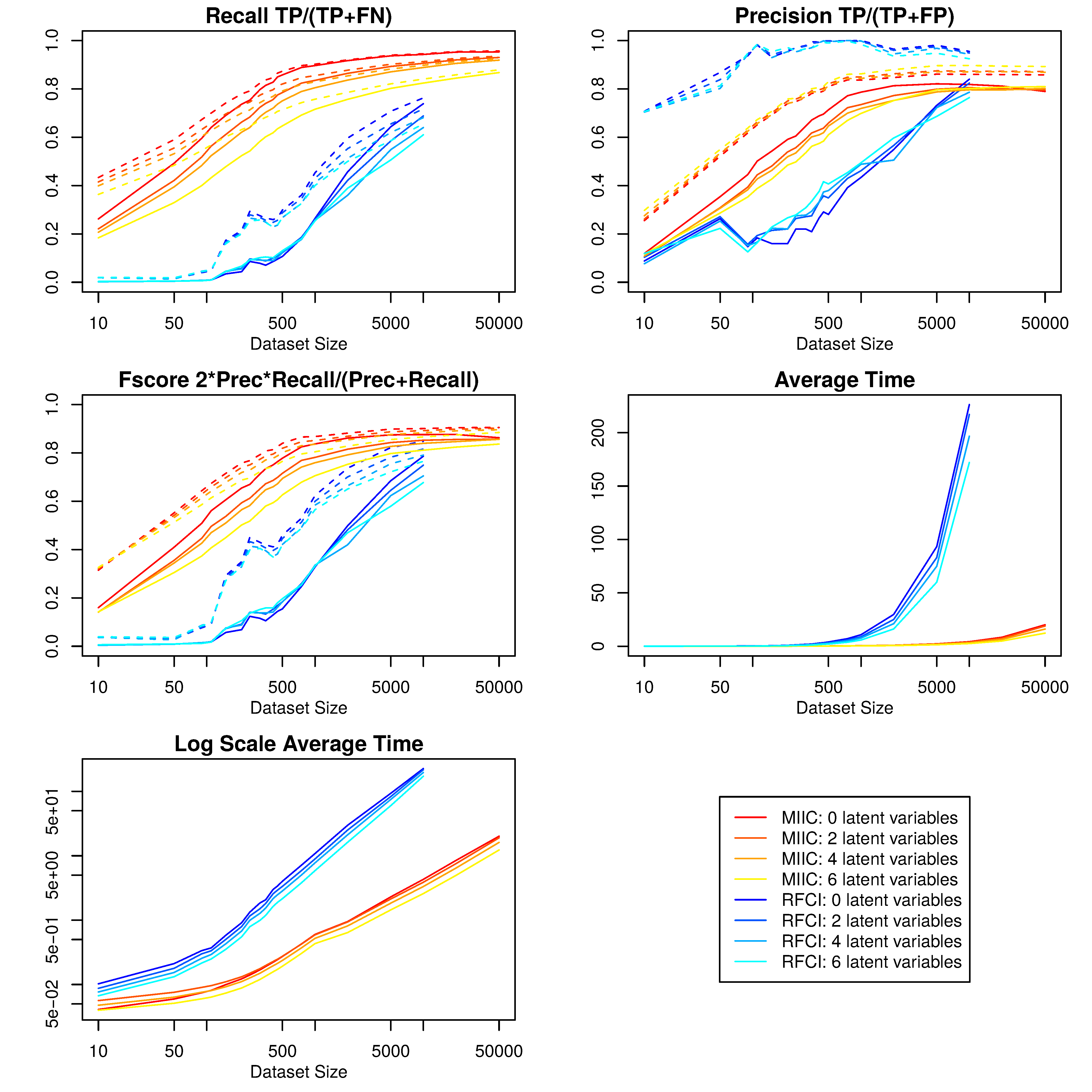

Supplement: S1 Fig — [37 nodes, 46 links, 509 parameters, Average degree 2.49, Maximum in-degree 4]. Precision, Recall, F-score and computing time for PAG skeletons (dashed lines) and PAGs including orientations (solid lines). The results are given for the miic algorithm (warm colors) compared to the RFCI algorithm [10] (cold colors) for 0, 2, 4 and 6 latent variables out of the 37 nodes. Computation times in log scale show a linear scaling in the limit of large datasets, τcpu ∼ N0.9, for the miic algorithm, and a stronger nonlinear increase, τcpu ∼ N1.5, with the RFCI algorithm. (TIFF) [file pcbi.1005662.s002.tiff]

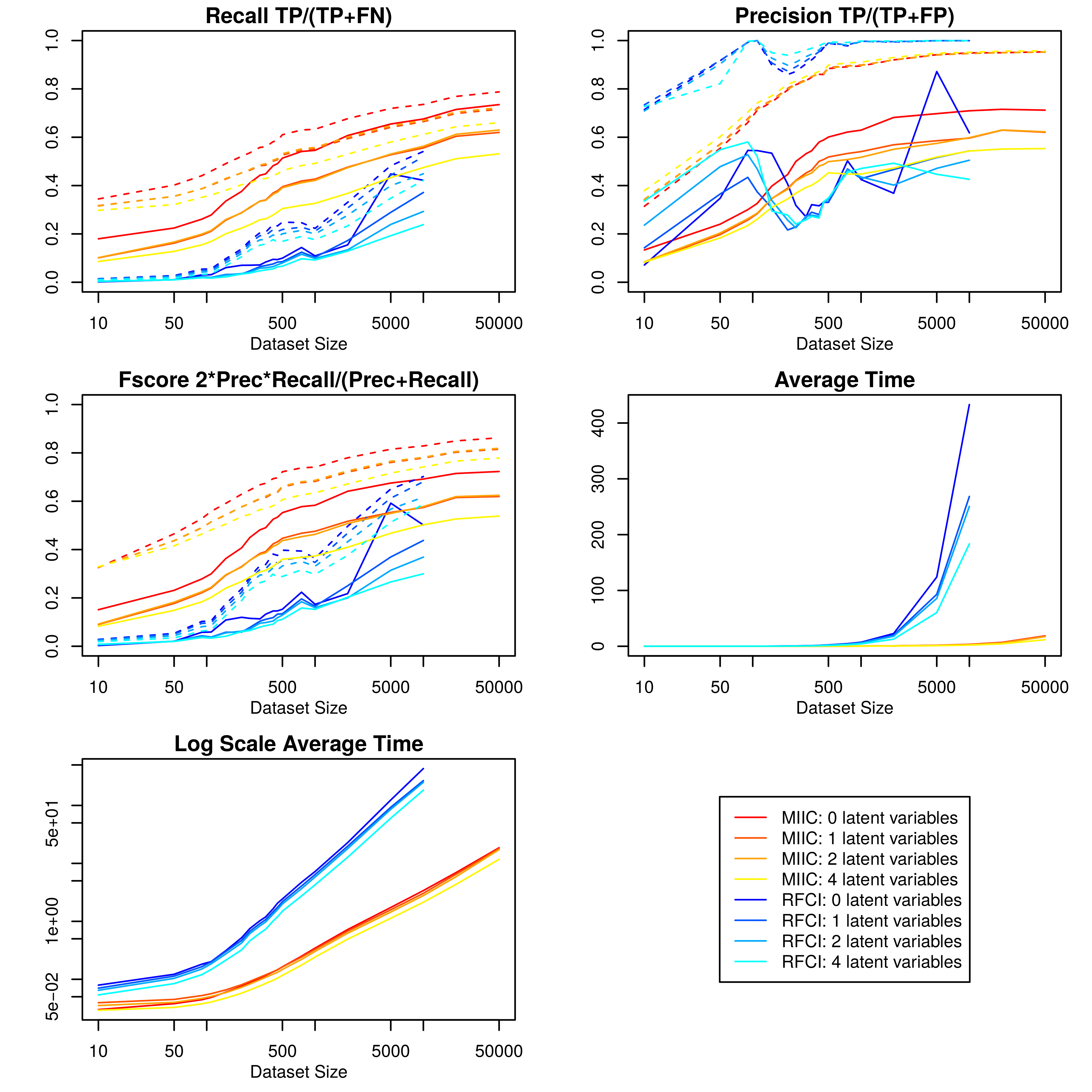

Supplement: S2 Fig — [27 nodes, 52 links, 984 parameters, Average degree 3.85, Maximum in-degree 3]. Precision, Recall, F-score and computing time for PAG skeletons (dashed lines) and PAGs including orientations (solid lines). The results are given for the miic algorithm (warm colors) compared to the RFCI algorithm [10] (cold colors) for 0, 1, 2, and 4 latent variables out of the 27 nodes. Computation times in log scale show a linear scaling in the limit of large datasets, τcpu ∼ N1.0, for the miic algorithm, and a stronger nonlinear increase, τcpu ∼ N1.7, with the RFCI algorithm. (TIFF) [file pcbi.1005662.s003.tiff]

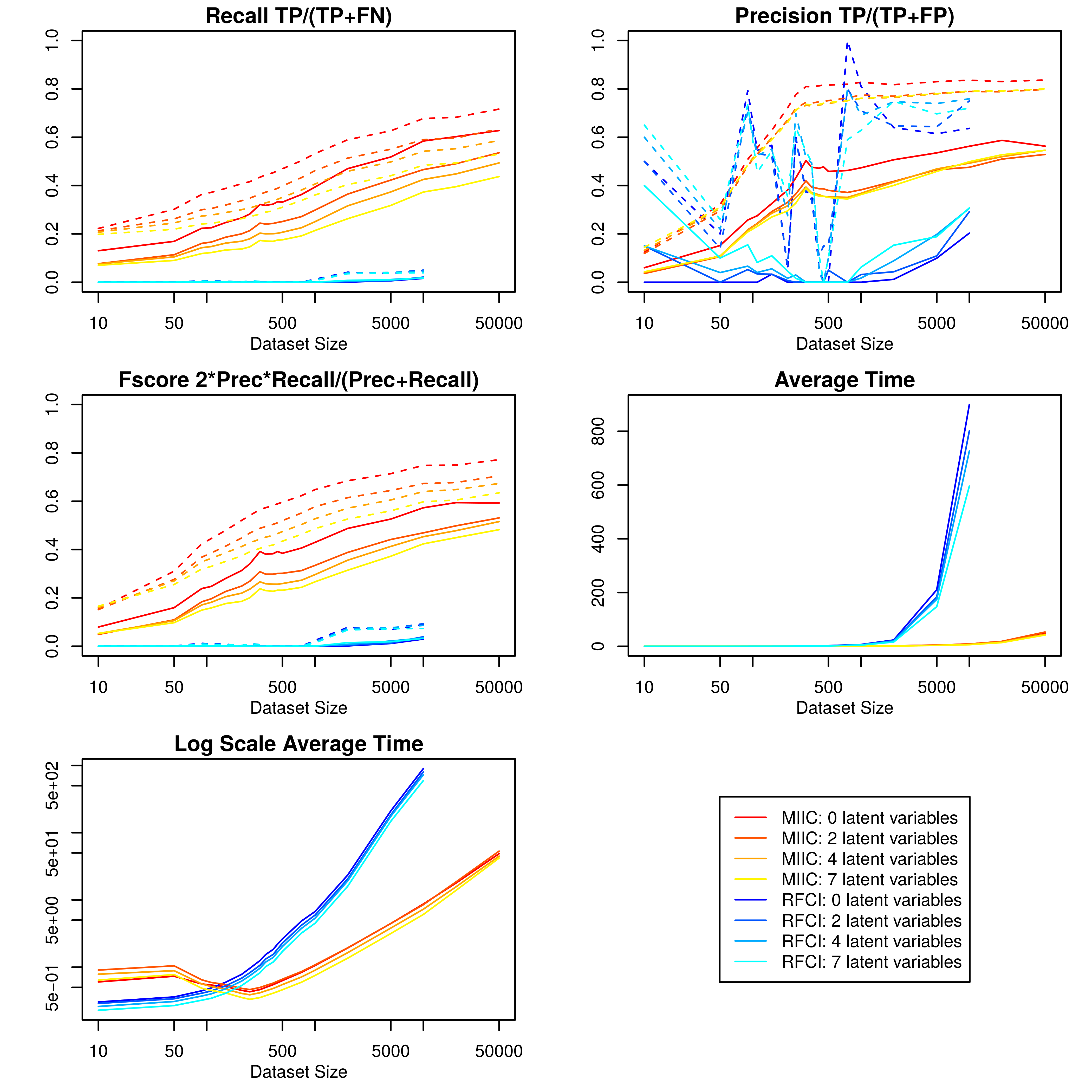

Supplement: S3 Fig — [48 nodes, 84 links, 114,005 parameters, Average degree 3.5, Maximum in-degree 4]. Precision, Recall, F-score and computing time for PAG skeletons (dashed lines) and PAGs including orientations (solid lines). The results are given for the miic algorithm (warm colors) compared to the RFCI algorithm [10] (cold colors) for 0, 2, 4 and 7 latent variables out of the 48 nodes. Computation times in log scale show a nearly linear scaling in the limit of large datasets, τcpu ∼ N1.1, for the miic algorithm, and a stronger nonlinear increase, τcpu ∼ N2.3, with the RFCI algorithm. (TIFF) [file pcbi.1005662.s004.tiff]

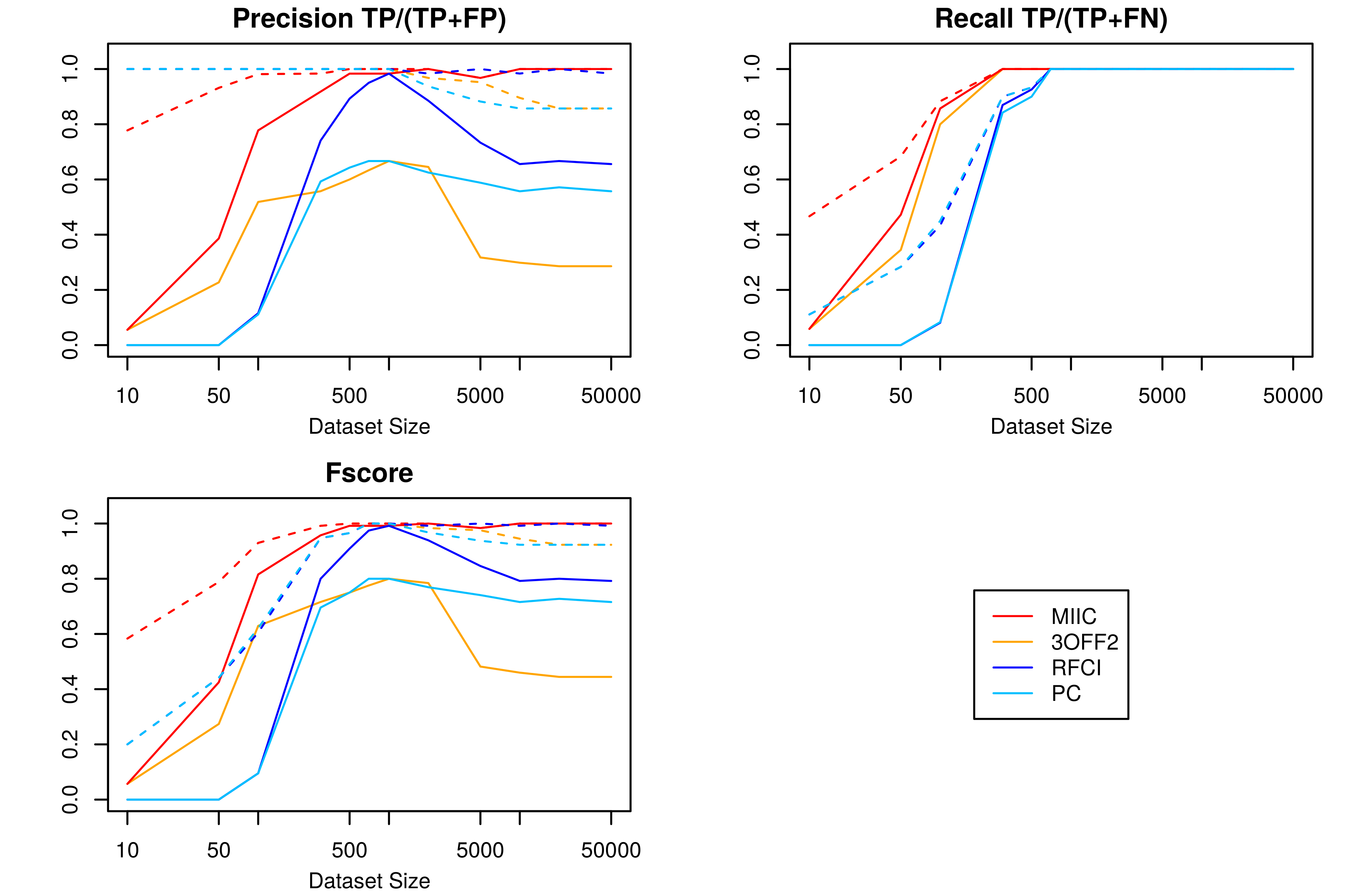

Supplement: S4 Fig — miic and RFCI [9, 10]versus 3off2 [19] and PC [7, 8, 25] reconstructions of Fig 1C network are performed from simulated data generated with Tetrad V, N = 10–50,000 samples. Precision, Recall and Fscore are given for skeleton (dashed lines) and PAG including orientations (solid lines). (TIFF) [file pcbi.1005662.s005.tiff]

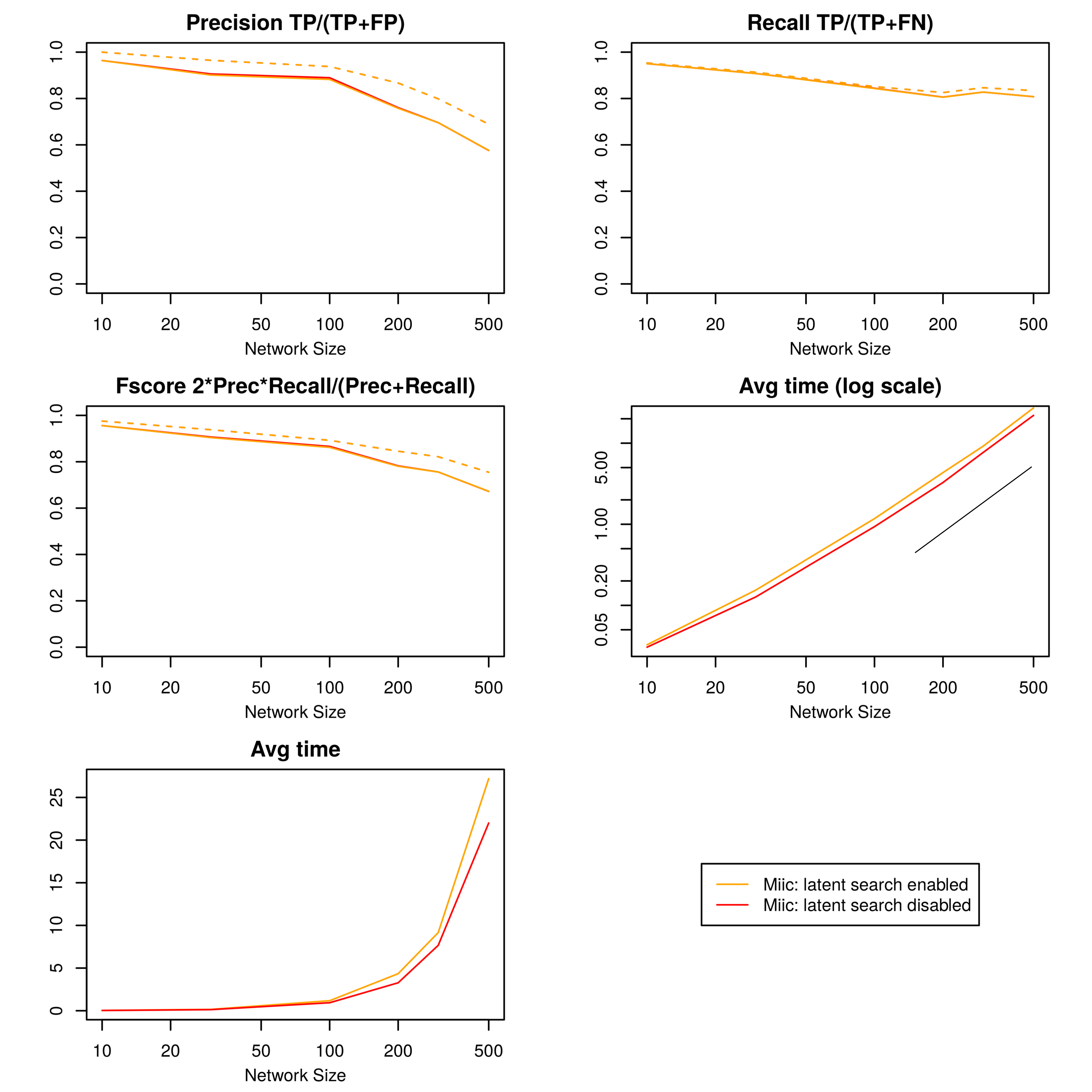

Supplement: S5 Fig — miic reconstruction of random networks of increasing size (P = 10–500 nodes) and fixed average degree 3 from N = 1,000 samples generated with Tetrad V. The average CPU time exhibits an optimal quadratic complexity in terms of network size, τcpu ∼ P2 (solid bar), with only a small time increase when considering latent variables (orange) as compared to excluding them (red). (TIFF) [file pcbi.1005662.s006.tiff]

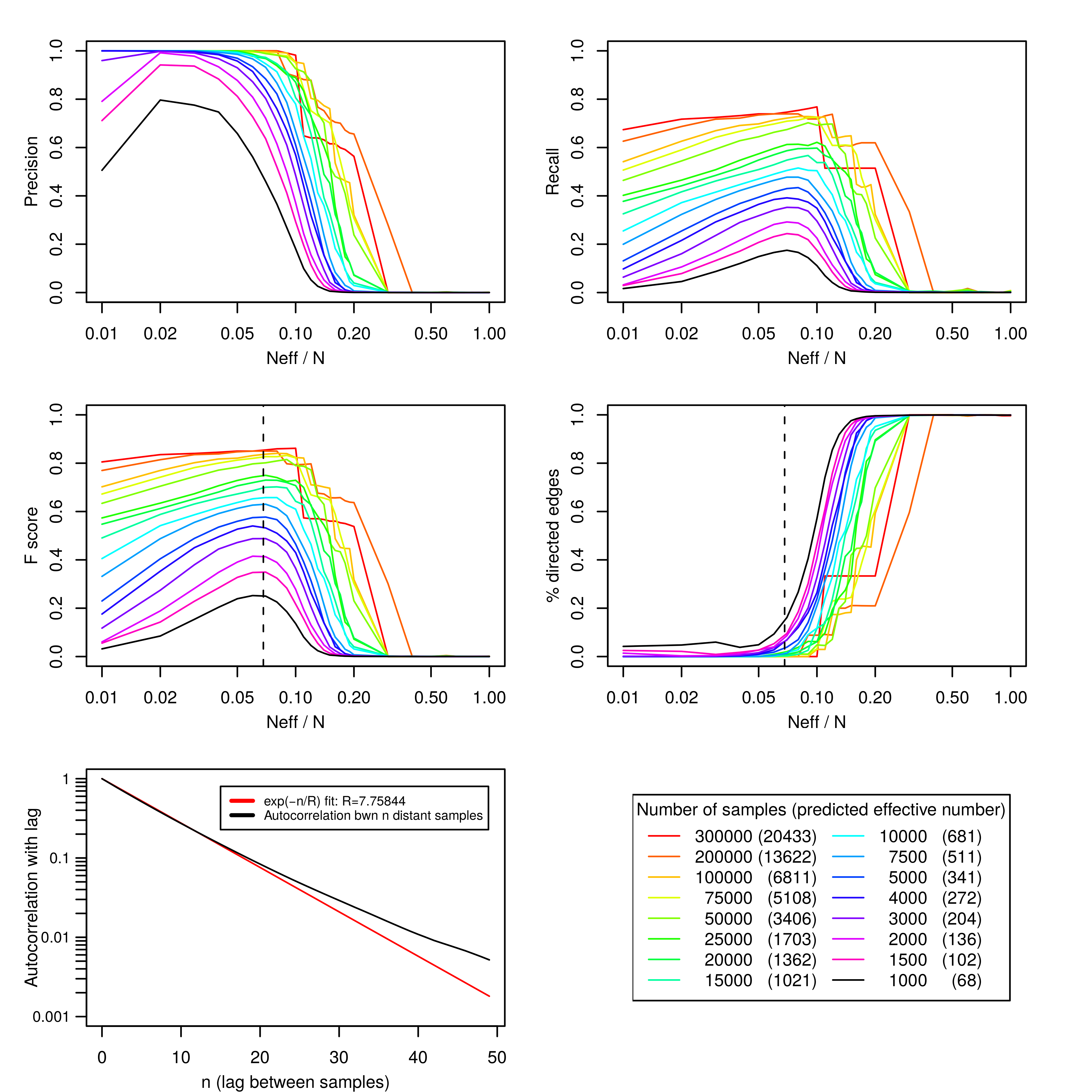

Supplement: S6 Fig — Precision, Recall, F-score, percentage of (wrongly) directed edges and decay of the autocorrelation function with lag between successive samples for N = 1,000 to 300,000 consecutive partially correlated samples (with predicted effective number of independent samples in brackets). Vertical dashed lines correspond to the predicted effective number of independent samples Neff*/N≃0.068, see Materials and methods. (TIFF) [file pcbi.1005662.s007.tiff]

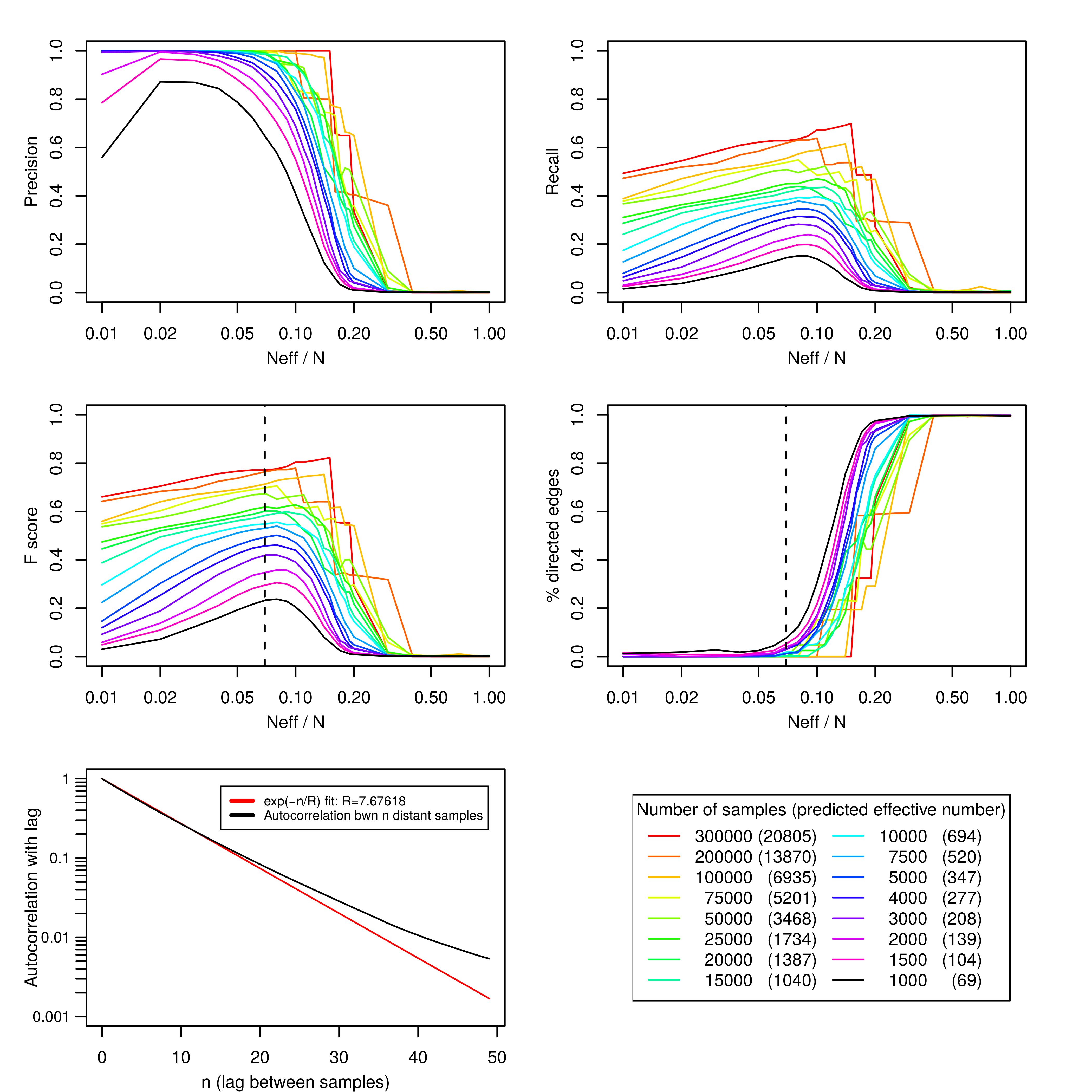

Supplement: S7 Fig — Precision, Recall, F-score, percentage of (wrongly) directed edges and decay of the autocorrelation function with lag between successive samples for N = 1,000 to 300,000 consecutive partially correlated samples (with predicted effective number of independent samples in brackets). Vertical dashed lines correspond to the predicted effective number of independent samples Neff*/N≃0.069, see Materials and methods. (TIFF) [file pcbi.1005662.s008.tiff]

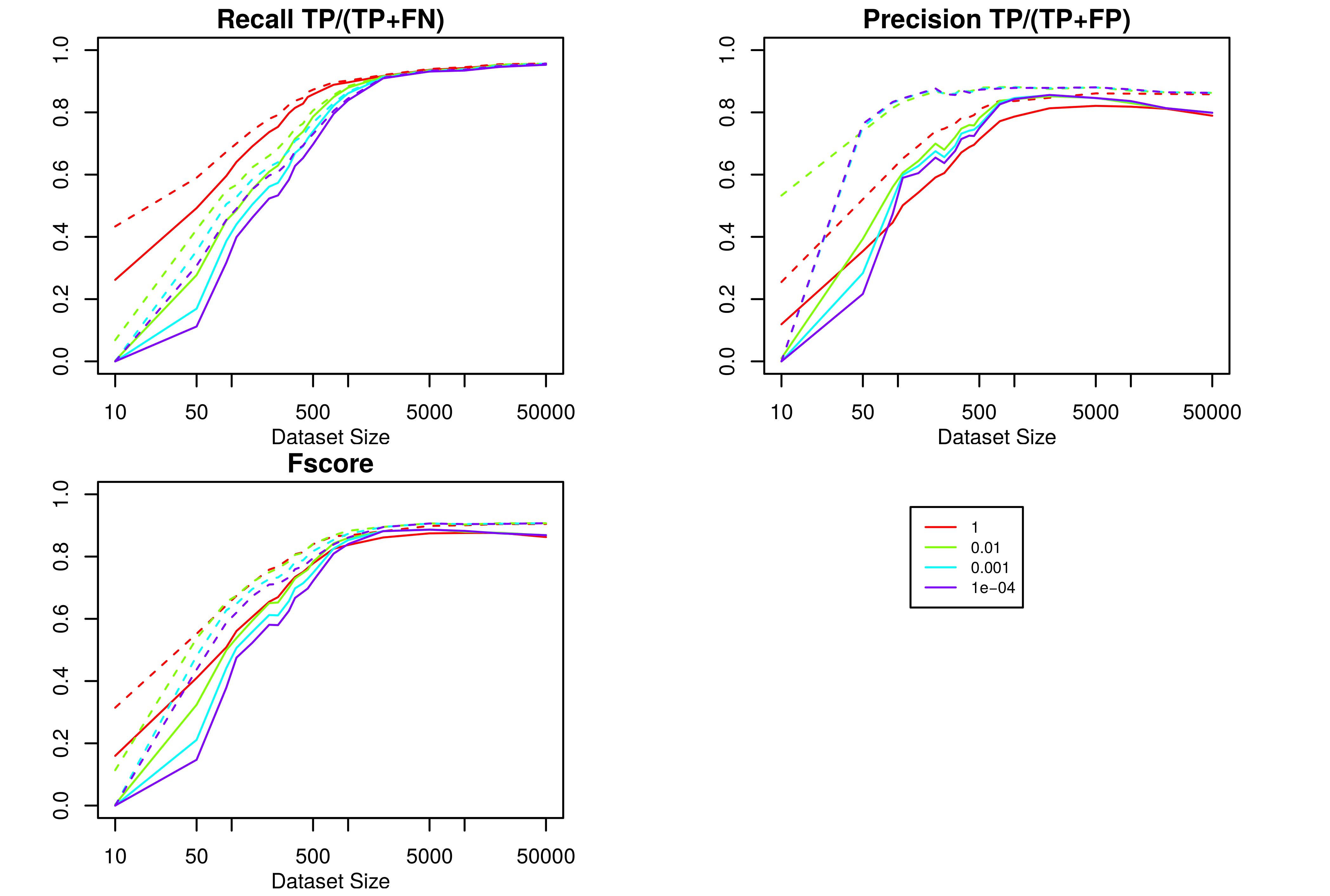

Supplement: S8 Fig — [37 nodes, 46 links, 509 parameters, Average degree 2.49, Maximum in-degree 4]. Precision, Recall, F-score and computing time for network skeleton (dashed lines) and oriented network CPDAG (solid lines) for a decreasing edge-specific confidence filtering, CXY = 1 (no filtering) 0.01, 0.001 and 0.0001. For sample size >100, confidence filtering of individual edges improves the precision (at the expense of recall) not only for the skeleton (dashed lines), as expected, but also for the oriented networks (solid lines). In addition, limited filtering, i.e. keeping edges with CXY < 10−3−10−2, tends to yield equivalent F-scores as unfiltered benchmark reconstructions. (TIFF) [file pcbi.1005662.s009.tiff]

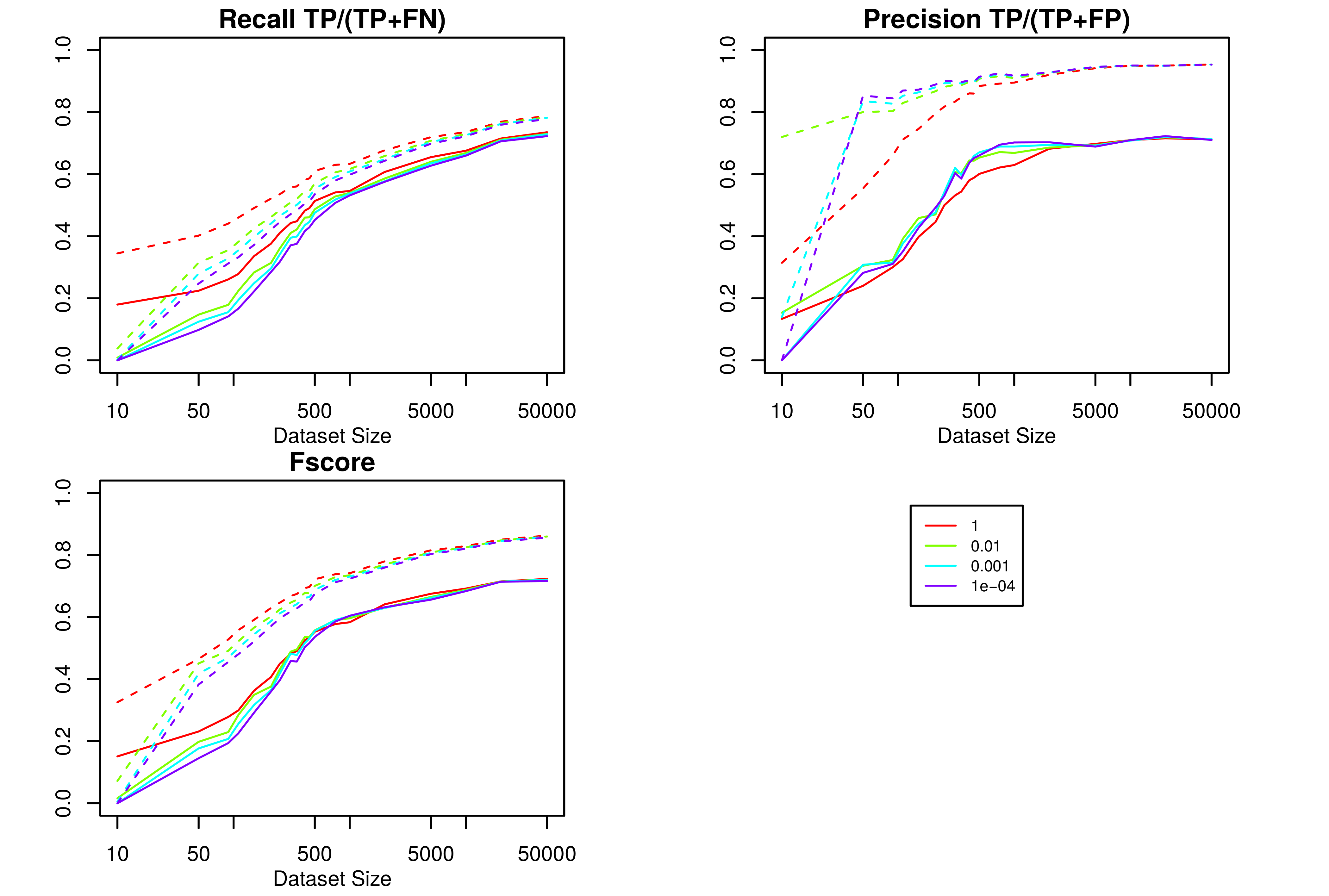

Supplement: S9 Fig — [27 nodes, 52 links, 984 parameters, Average degree 3.85, Maximum in-degree 3]. Precision, Recall, F-score and computing time for network skeleton (dashed lines) and oriented network CPDAG (solid lines) for a decreasing edge-specific confidence filtering, CXY = 1 (no filtering) 0.01, 0.001 and 0.0001. For sample size >100, confidence filtering of individual edges improves the precision (at the expense of recall) not only for the skeleton (dashed lines), as expected, but also for the oriented networks (solid lines). In addition, limited filtering, i.e. keeping edges with CXY < 10−3−10−2, tends to yield equivalent F-scores as unfiltered benchmark reconstructions. (TIFF) [file pcbi.1005662.s010.tiff]

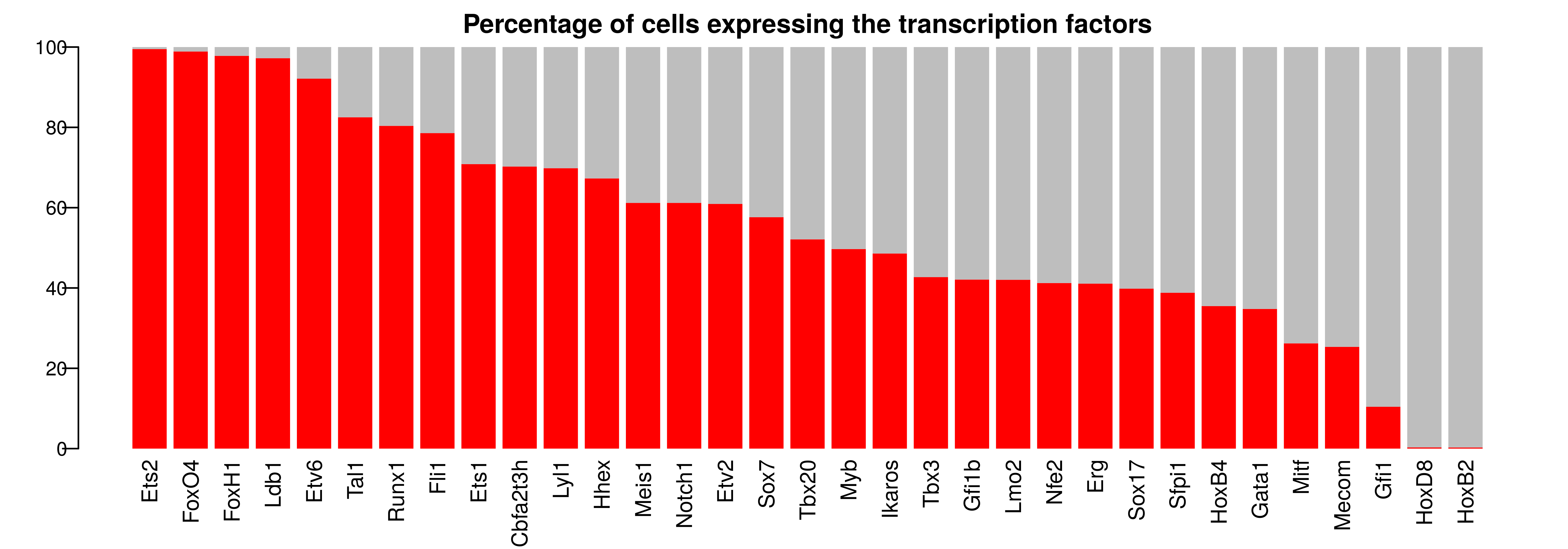

Supplement: S10 Fig — Expression data on the 33 TFs are obtained from [24]. Percentage of samples with expressed genes (red) and non-expressed genes (gray). (TIFF) [file pcbi.1005662.s011.tiff]

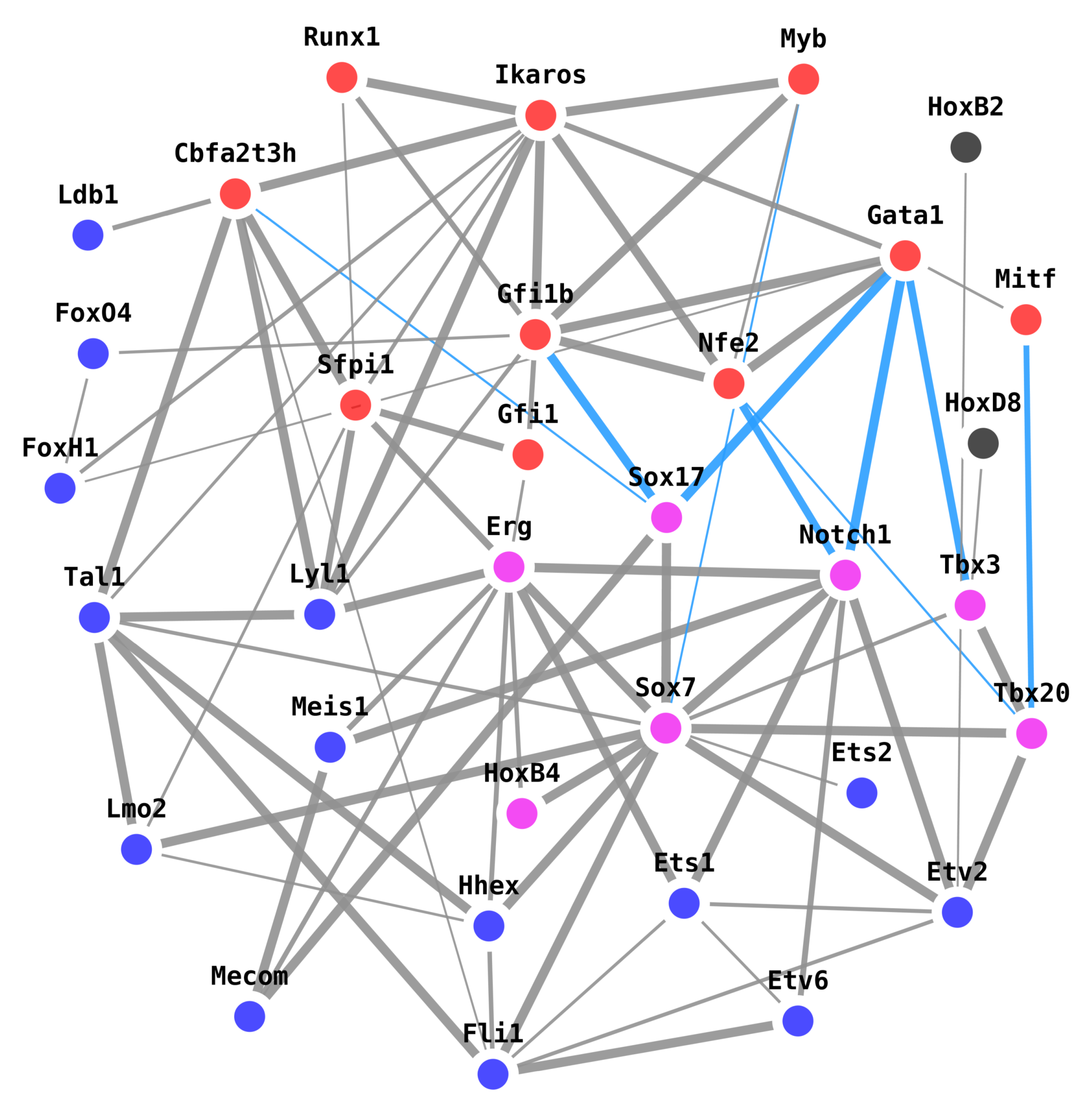

Supplement: S11 Fig — Hematopoietic / endothelial gene expression data in 3,934 single cells from mouse embryos [24]. 7 out of 82 edges (8.5%) with CXY > 10−3 have been filtered in Fig 2D (blue edges correspond to anti-correlations). (TIFF) [file pcbi.1005662.s012.tiff]

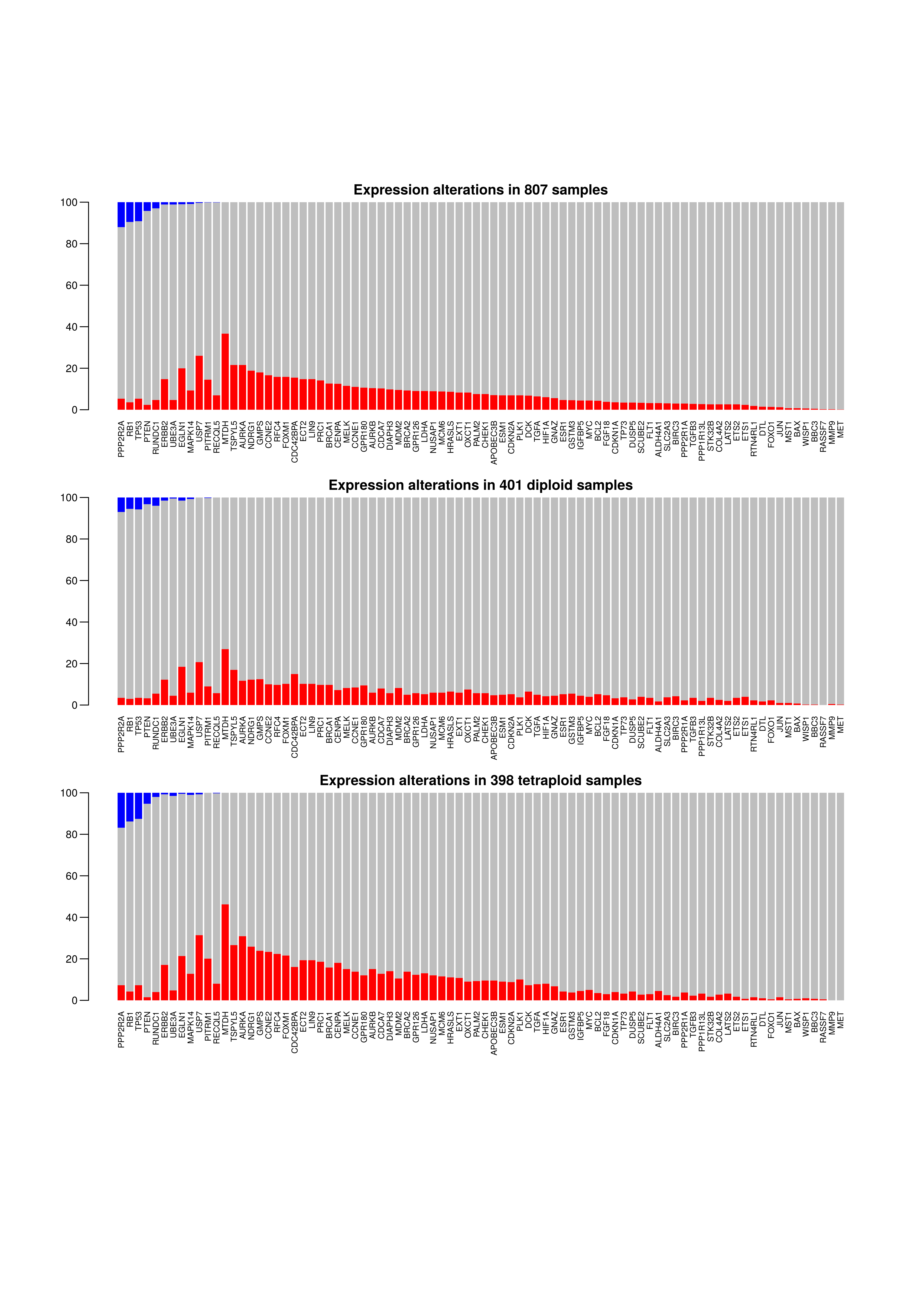

Supplement: S12 Fig — Percentage of samples with normalized over-expression (red), normalized under-expression (blue) and unchanged normalized expression (gray) for each gene based on COSMIC. (TIFF) [file pcbi.1005662.s013.tiff]

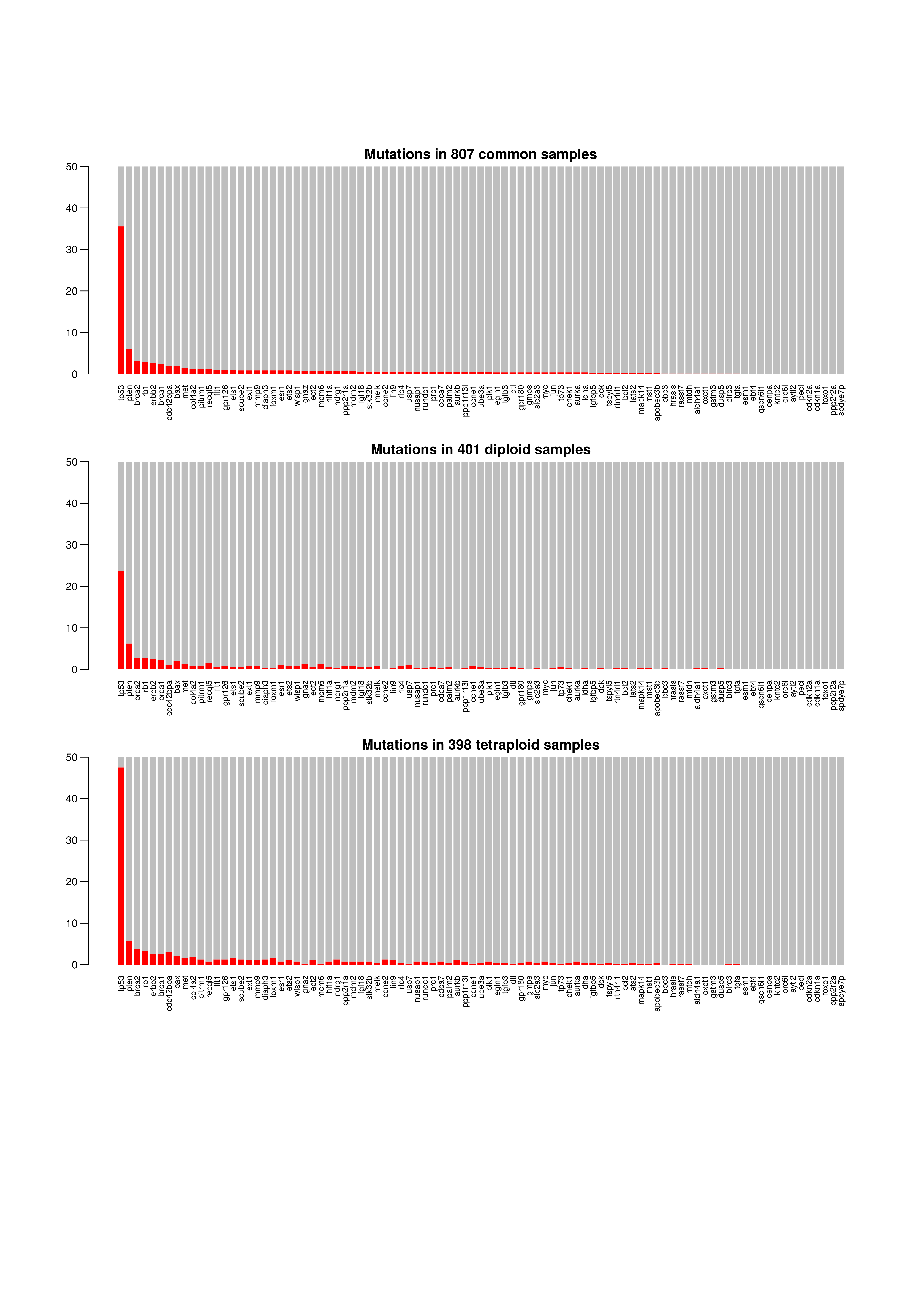

Supplement: S13 Fig — Percentage of mutated samples (red) for each gene. (TIFF) [file pcbi.1005662.s014.tiff]

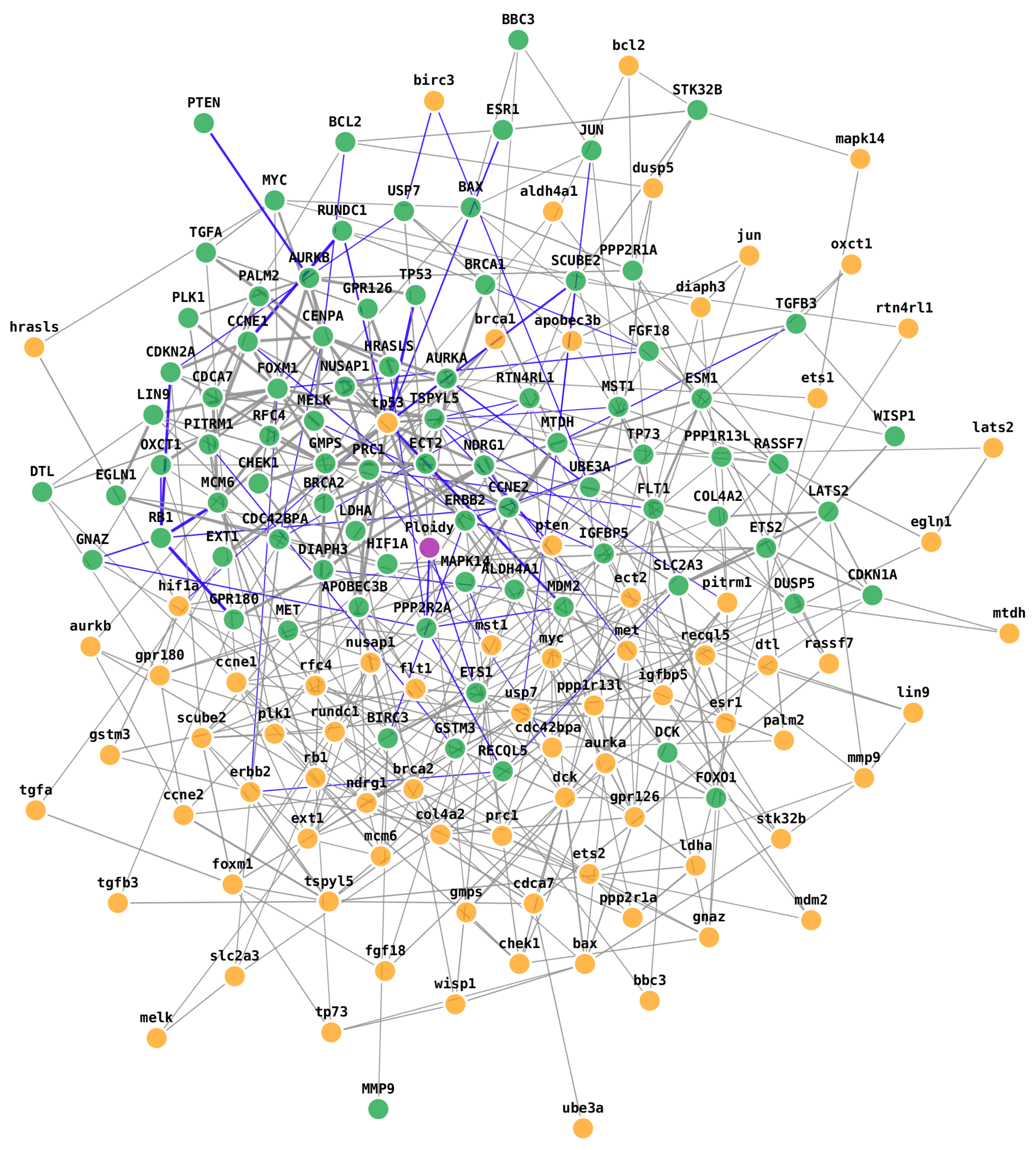

Supplement: S14 Fig — Due to the limited numbers of samples (N = 807) and recurrent gene mutants (Figure -figure supplement 2), most gene mutations (yellow) are not confidently linked to any altered expression levels (green) and have been filtered in the high confidence network Fig 3D (CXY < 10−3), with the notable exceptions of TP53 and RB1 mutations, which have a significant impact on gene expressions, Fig 3D, see main text (blue edges correspond to anti-correlations). (TIFF) [file pcbi.1005662.s015.tiff]

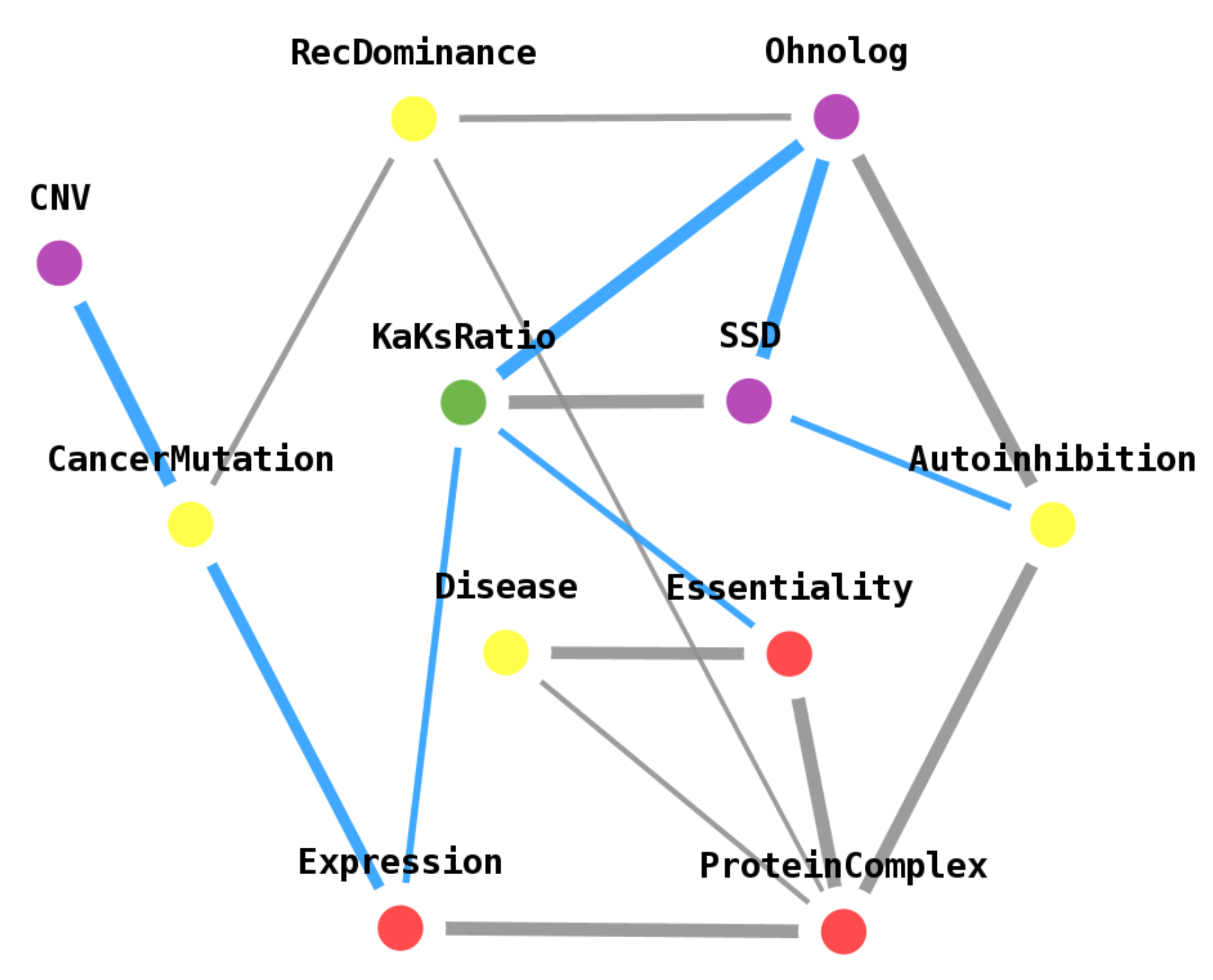

Supplement: S15 Fig — Genomic data for the 20,415 human coding genes is provided in S1 Data. The only edge with confidence ratio CXY > 10−3 is RecDominance − ProteinComplex with CXY = 0.25 (blue edges correspond to anti-correlations). (TIFF) [file pcbi.1005662.s016.tiff]
