# Supplementary material for: Learning causal networks with latent variables from multivariate information in genomic data
Source: PLoS Comput Biol. 2017 Oct 2;13(10):e1005662. doi: 10.1371/journal.pcbi.1005662 (PMC5685645; doi:10.1371/journal.pcbi.1005662)
Supplement: S1 Software — miic software is provided in two formats: an R-package to be used in the R environment, and miic and FCI executables, which were used for all benchmarks included in the paper. (ZIP) [file pcbi.1005662.s017.zip › miic_fci_executables.zip!/miic_software/miic_user-guide.pdf]

Miic User Guide

(using miic and FCI)

September 28, 2016

1 Package and scripts

The main folder contains the scripts and source code for the reconstruction of networks starting from observation data. The directories are organized as follows:

```
/
├── common
│   ├── miic.R
│   ├── gmPlot.R
│   └── gmSummary.R
├── sharedLib
├── data
│   └── some data input/output...
├── miic
│   └── miic sources and executables
├── confidence_tool
│   └── confidence cut sources and executables
└── fci
    └── fci scripts
```

2 Package requirements

To launch the miic.R script you need to have R installed on your machine (R release $\geq$ 3.3.1), along with some packages that are available in the CRAN repository.

Rpackages getopt, plotrix, igraph, ppcor, bnlearn, pcalg

3 Calling the inference methods with `miic.R`

You can call the inference methods through the *miic.R* script.

Overview

main *~/common/miic.R*

lib *~/common/lib/...*

Arguments *(mandatory: *)*

-i * file path of the input dataset[1]

-o * directory path for the output of the inference method[2]

-m inference method (*miic*, *fci*)
default: *miic*

-d steps to perform ('1,2,3,4' or '1,2' or '1,3' *etc...*)
default: '1,2,3,4'[3]

-p parameters for the inference method (see the following subsections). The value expected here is of type character: '$param_1$:$value_1$,*etc...*'

-t file path to the true edges; used during the *summary* step[4]

-l file path to the layout of each vertex; used during the *plot* step[5]

-c if given, edges will be filtered according to their confidence ratio. It needs two parameters, described in the part "Option '-c' for `miic`".

-s if given, this file provides an ordering of each variable categories for the calculation of the sign of the edges. The signs are calculated using Spearmans partial correlation coefficient. The ordering file is automatically generated if not provided by the user and can be edited to properly reorder the variable categories. See an example of stateOrder file in the data directory.

A full example (common/miic.R):
*Rscript **miic.R** **-i** ../data/alarm1000samples.txt **-o** ../data/alarmNetwork **-m** miic **-p** cpx:nml,efn:1000,lat:yes,prg:yes **-c** csh:100,ccr:0.01 **-l** ../data/alarmLayout.txt **-s** ../data/alarmStateOrder.tsv*

When calling the available inference methods with *miic.R*, the '-p' option can be used to indicate the chosen parameters. The value expected for this option is

[1]The input dataset should be a tab separated table, with column names but no row names. Missing values should be indicated with *NA*. Each column corresponds to a categorized variable and each row to one sample.

[2]To prevent from overwriting existing results, if the output directory already exists, the skeleton inference step returns a message and stops.

[3](1) skeleton, (2) probabilistic orientation, (3) summary, (4) plot

[4]The true edges file has two space-separated columns. Each line corresponds to one true edge. The orientation is $col1 \rightarrow col2$.

[5]The layout file has three tab separated columns, the first column being optional. Each line corresponds to the (x, y) coordinates of each vertex. The first column can contain the label of the vertex as indicated in the colnames of the input dataset table. The order in which the coordinates are given also corresponds to the order of the colnames of the input dataset table.

of type character: '$param_1$:$value_1$,$param_2$:$value_2$ *etc...*'. The possible $param_i$ and $value_i$ for each method are detailed in the following subsections.

Note: the root folder of the provided source includes a *permission.sh* script that enables unix users to easily grant permissions to all `miic` executable files. In this root folder where *permission.sh* is located, change the executable permission as *(sudo) chmod a+x permission.sh* and run the script as *(sudo) ./permission.sh* (using the *sudo* command if required by the user OS settings).

Option '-p' for `MIIC`

cpx formula used to compute the complexity term ['mdl'[6] or 'nml'[7]]
default: nml (*Ex.: -p '...,cpx:mdl,...'*)

lat should the network be reconstructed under the hypothesis that some variables might not be observed? ['yes' or 'no']
default: no (*Ex.: -p ...,lat:yes,...*)

prg should the network be oriented using the propagation rule? ['yes' or 'no']
default: yes (*Ex.: -p ...,prg:yes,...*)

efn number of uncorrelated samples
default: number of rows of the input dataset (*Ex.: -p ...,efn:1000,...*)

A full '-p' example for MIIC (common/miic.R):
Rscript ***miic.R -i*** *../data/alarm1000samples.txt* ***-o*** *../data/alarmNetworkMiic* ***-m*** *miic* ***-p*** *cpx:nml,efn:1000,lat:yes,prg:yes* ***-c*** *csh:100,ccr:0.01* ***-l*** *../data/alarmLayout.txt*

Option '-p' for `FCI`

aph α significance level for the statistical independence tests[8]
default: 0.01 (*Ex.: -p ...,aph:0.001,...*)

cit type of statistical independence test ['*gaussCItest*', '*dsepTest*', '*disCItest*', '*binCItest*']
default: gaussCItest (*Ex.: -p ...,cit:disCItest,...*)

skm choose the original PC or the order-independant version ['*stable*', '*original*', '*stable.fast*']
default: stable (*Ex.: -p ...,skm:original,...*)

typ which type of fci variant should be used[9] ['*normal*', '*rapid*', '*adaptive*', '*anytime*']
default: normal (*Ex.: -p ...,upd:rapid,...*)

[6]Maximum description length
[7]Normalized Maximum Likelihood
[8]refer to the documentation of the R package 'pcalg' for details
[9]RFCI corresponds to 'rapid'

con set the state of the conservative rule [‘*TRUE*’, ‘*FALSE*’]
default: FALSE (*Ex.: -p ...,con:TRUE,...*)

maj set the state of the majority rule [‘*TRUE*’, ‘*FALSE*’]
default: FALSE (*Ex.: -p ...,maj:TRUE,...*)

A full ‘-p’ example for FCI (common/miic.R):
Rscript ***miic.R -i*** *../data/alarm1000samples.txt* ***-o*** *../data/alarmNetworkFci* ***-m*** *fci*
-p *aph:0.000001,cit:disCItest,skm:stable,typ:normal,con:FALSE,maj:FALSE*
-l *../data/alarmLayout.txt*

Option ‘-c’ for `miic`

csh number of random shuffling of the input dataset, in order to get the random mutual information between `miic` inferred edges (*Ex.: -c ...,csh:10,...*)

ccr confidence ratio used as a threshold for filtering the edges. (*Ex.: -c ...,ccr:0.01,...*)

A full ‘-c’ example for MIIC (common/miic.R):
Rscript ***miic.R -i*** *../data/alarm1000samples.txt* ***-o*** *../data/alarmNetwork* ***-m*** *miic*
-p *cpx:nml,efn:1000,lat:yes,prg:yes* ***-c*** *csh:100,ccr:0.01* ***-l*** *../data/alarmLayout.txt*

4 Viewing inferred networks

The inferred networks can be viewed in pdf format (automatically generated with igraph (`http://www.igraph.org/`) or manipulated for better display using cytoscape (`http://www.cytoscape.org/`) (graphml format). The files are located in the following directories:

- Unfiltered network: the pdf ‘edgesList.miic.summary.plot_confidence.pdf’ (only with the -m miic option), ‘edgesList.miic.summary.plot_pCor.pdf’ and graphml ‘[output name].graphml’ files are located in the output directory set by the -o entry in the command line
- Filtered networks (using -c option with miic): pdf and graphml files are in the subdirectory ‘shuffle [cshValue]/filtered network [ccrValue]’, which can be found in the output directory set by the -o entry in the command line. The output folder name in this case is ‘filtered network [ccrValue]’.

In order to visualize the network in a correct, pleasant and interactive way, we recommend the utilization of Cytoscape tool, version 3.1.0 or later. Cytoscape is available for Windows, Linux and OS X. Visualizing `miic` networks with Cytoscape requires to go through the following steps:

a. Import the network: File⇒Import⇒Network⇒File, and select the graphml file in the created output directory

b. Import the style: File⇒Import⇒Styles, and select the miic_style.xml file present at the root of the provided source directory

c. Select the loaded style: under the Style panel present in Control Panel select the miic_style

5 Output files

The execution of miic will create the output files described below, which will be located inside the output folder.

5.1 adjacencyMatrix.miic.orientProba.txt, adjacencyMatrix.miic.txt

The adjacency matrix is a square matrix used to represent the inferred graph. The entries of the matrix indicate whether pairs of vertices are adjacent or not in the graph. The matrix can be read as a (row, column) set of couples where the row represents the source node, X, and the column the target node, Y. Since miic can reconstruct mixed networks (directed, undirected and bidirected), we will have a different digit for each case:

- 1: XY edge is undirected as $X - Y$
- 2: XY edge is directed as $X \rightarrow Y$
- -2: XY edge is directed as $X \leftarrow Y$
- 6: XY edge is bidirected as $X \leftrightarrow Y$

5.2 edgesList.miic.summary.txt

The summary file is a table containing detailed informations about inferred and not inferred edges.

- **x**: X node
- **y**: Y node
- **type**: inferred (P), not inferred (N). If the true edges file is also given, P will become TP (true positive) or FP (false positive), and N will become TN (true negative) of FN (false negative). This information is only taking into account at the skeleton level
- **ai**: contains the comma separated list of the best contributing ancestor nodes, $\{A_i\}$.
- **info**: the remaining information between X and Y after conditioning on $\{A_i\}$, *i.e.* $I(X;Y|\{A_i\})$

- **cplx**: the NML or MDL complexity value $k_{X;Y|\{A_i\}}$
- **Nxy_ai**: the number of samples for which the values for variables X,Y and $\{A_i\}$ are available (no 'NA')
- **(-log) confidence**: $-logP_{XY} = NI(X;Y|\{A_i\}) - k_{X;Y|\{A_i\}} = NI'(X;Y\{A_i\})$ value. It is a way to quantify the strength of the edge XY
- **confidenceRatio**: if -c option is provided, the ratio between the probability to "reject" edge XY in the dataset *versus* the mean probability to do the same in randomised datasets
- **infOrt**: the orientation of the edge XY. It is the same value as in the adjacency matrix at row x and column y
- **trueOrt**: the orientation of the edge XY present in the true edges file (if provided with -t option)
- **isOrtOk**: information about the consistency of the inferred graph's orientations with the reference graph given (with -t).

  ⇒ Y: the orientation is consistent

  ⇒ N: the orientation is not consistent
- **sign**: sign of the partial correlation for edge XY conditioned on $\{A_i\}$
- **diff**: value of the partial correlation for edge XY conditioned on $\{A_i\}$

5.3 [output name].graphml

GraphML is a comprehensive and easy-to-use file format for graphs. It consists of a language core to describe the structural properties of a graph and a flexible extension mechanism to add application-specific data. This file allows users to visualize the network with Cytoscape directly on their personal computers. In order to visualize the network correctly, users should also import the miic_style file.

5.4 execTime.miic.skeleton.txt, execTime.miic.orient.skeleton.txt

These files contain informations about the running time for the skeleton and the orientation steps of the execution.

5.5 edgesList.miic.summary.plot_confidence.pdf, edgesList.miic.summary.plot_pCor.pdf

These files contain the plot of the reconstructed network reporting respectively the confidence or the correlation value as edge strength. The plots are made using the igraph package.
